# *regeneration factors expressed on myeloid* expression in macrophage-like cells is required for tail regeneration in *Xenopus laevis* tadpoles

**DOI:** 10.1242/dev.200467

**Published:** 2023-07-31

**Authors:** Momoko Deguchi, Taro Fukazawa, Takeo Kubo

**Affiliations:** Department of Biological Sciences, Graduate School of Science, University of Tokyo, Bunkyo-ku, Tokyo, 113-0033, Japan

**Keywords:** *Xenopus laevis*, Tail regeneration, Leukocyte, Reparative myeloid, Macrophage

## Abstract

*Xenopus laevis* tadpoles can regenerate whole tails after amputation. We have previously reported that *interleukin 11* (*il11*) is required for tail regeneration. In this study, we have screened for genes that support tail regeneration under Il11 signaling in a certain cell type and have identified the previously uncharacterized genes *Xetrov90002578m.L* and *Xetrov90002579m.S* [referred to hereafter as *regeneration factors expressed on myeloid.L* (*rfem.L*) and *rfem.S*]. Knockdown (KD) of *rfem.L* and *rfem.S* causes defects of tail regeneration, indicating that *rfem.L* and/or *rfem.S* are required for tail regeneration. Single-cell RNA sequencing (scRNA-seq) revealed that *rfem.L* and *rfem.S* are expressed in a subset of leukocytes with a macrophage-like gene expression profile. KD of *colony-stimulating factor 1* (*csf1*), which is essential for macrophage differentiation and survival, reduced *rfem.L* and *rfem.S* expression levels and the number of *rfem.L-* and *rfem.S*-expressing cells in the regeneration bud. Furthermore, forced expression of *rfem.L* under control of the *mpeg1* promoter, which drives *rfem.L* in macrophage-like cells, rescues *rfem.L* and *rfem.S* KD-induced tail regeneration defects. Our findings suggest that *rfem.L* or *rfem.S* expression in macrophage-like cells is required for tail regeneration.

## Teaser

**Summary:** *regeneration factors expressed on myeloid* genes induced in the regeneration bud are required for the regeneration-promoting function of macrophages in *Xenopus laevis* tail regeneration.

## INTRODUCTION

*Xenopus laevis* tadpoles can regenerate whole tails with muscles, notochord and spinal cord in one week (Beck et al., 2009; Slack et al., 2008). It has been suggested that, after tail amputation, lineage-restricted progenitor cells, which are produced from stem cells of each type of tissue differentiate into their own tissues to form a regenerated tail (Cesare and Slack, 2004). In our previous study, we found that *il11*, the expression of which is induced after tail amputation, is required and sufficient for producing several types of progenitor cells; thus, it is essential for tail regeneration (Tsujioka et al., 2017). We found that even progenitor cells in which *il11* receptors are knocked out contribute to the formation of the regenerated tail, suggesting that the progenitor cell induction function of Il11 is elicited in a non-cell autonomous manner (Suzuki et al., 2022). These findings led us to hypothesize that Il11 induces progenitor cells via a specific cell type that supports tail regeneration in response to Il11. In this study, we aim to identify the putative cell type by screening genes that function in the putative cells.

## RESULTS AND DISCUSSION

### Expression of *rfem.L* and *rfem.S* is induced by tail amputation

To identify the putative supporting cells, we searched for genes thought to function in the cells using published RNA-sequencing (RNA-seq) data of amputated tails from *il11* knockdown (KD) tadpoles (Tsujioka et al., 2017) in order to screen for genes that function in response to Il11. We obtained 1266 genes that were downregulated in *il11* KD groups. Next, to screen for genes that function during tail regeneration but not during development, we analyzed the RNA-seq data of proliferating cells from developing tailbuds and proliferating cells and non-proliferating cells from regeneration buds (Tsujioka et al., 2015), and narrowed down the candidates to seven genes that are rarely expressed in the proliferating cell fraction of the tailbud. Next, to screen for genes expressed in the putative supporting cells other than the proliferating progenitor cells, we further narrowed down the candidates to those with higher expression in the non-proliferating cell fraction than in the proliferating cell fraction and identified four genes (Table S1). Among them, we focused on *Xetrov90002578m.L* and *Xetrov90002579m.S* because their functions have not been reported and they are rarely expressed in early development (Session et al., 2016) (Fig. S1A). *X. laevis* is an allotetraploid species and many genes have homologous genes on L and S chromosomes (Session et al., 2016). We identified *Xetrov90002578m.L* and *Xetrov90002579m.S* to be homologous, as they have very similar sequences and are located on homologous L and S chromosomes, respectively, with conserved synteny (Fig. S1B). Hereafter, we refer to them as *regeneration factors expressed on myeloid.L* (*rfem.L*) and *rfem.S*, respectively. There is a putative *rfem.L* and *rfem.S* orthologue in *Xenopus tropicalis* (accession number KAE8633781), a closely related species of *X. laevis,* but no orthologues have been identified in fish and rodents. The functions of Rfem.L and Rfem.S were not predictable from their amino acid sequences because there were no remarkable domains, although they showed weak similarity to γ-crystallin, and have no putative signal peptide sequences, suggesting their intracellular function.

**Fig. 1.**
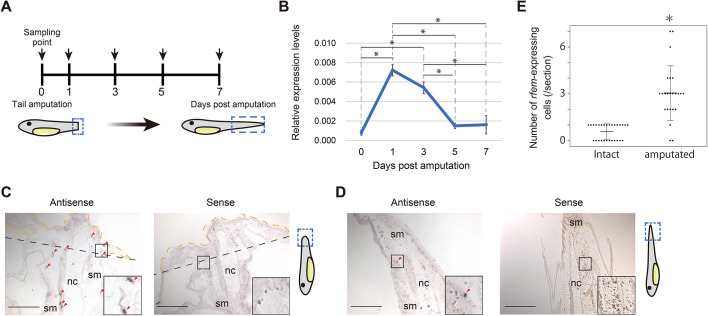
***rfem.L* and *rfem.S* expression is upregulated after tail amputation and is detected in scattered cells in the amputated tails.** (A) Experimental design of expression analysis. Tail stumps (0 dpa) and regenerating tails (1 to 7 dpa) were sampled at the time-points indicated with arrows. (B) Relative expression levels of *rfem.L* and *rfem.S* in the tail stumps/regenerating tails. Vertical axes represent relative expression of *rfem.L* and *rfem.S* normalized to that of *ef1a*. Data are mean±s.d., *n*=4. *P<0.05, Tukey-Kramer's test. (C,D) Representative images of *in situ* hybridization of *rfem.L* and *rfem.S* with (left) the antisense probe and (right) the sense probe on sagittal sections of tails from (C) 3 dpa or (D) intact tadpoles. Posterior is upwards and dorsal is leftwards. Red arrowheads indicate representative signals. Black dashed lines indicate the amputation position. Areas enclosed with orange dashed lines in C indicate the regeneration buds. Insets in the figures are magnified images of the outlined areas. Blue dashed lines in the schematic diagrams outline the location of the sections. nc, notochord; sm, skeletal muscle. Scale bars: 200 μm. (E) Number of *rfem*-expressing cells in sections of intact (28 sections from nine tadpoles) or 1 dpa (24 sections from nine tadpoles) tails. Data are mean±s.d., *P<0.05, Welch's *t*-test.

Temporal expression analysis of tail stumps (Fig. 1A) revealed that the expression level of *rfem.L* and *rfem.S* at the tail stump became significantly higher at 1 to 3 days post amputation (dpa) than that at 0 dpa (Fig. 1B). The temporal expression pattern correlated well with the formation of the regeneration bud, which forms within 2 dpa (Beck et al., 2003). Expression of *rfem.L* and *rfem.S* is decreased at 5 to 7 dpa (Fig. 1B), when tail regeneration is almost complete. These results indicate that the expression of *rfem.L* and *rfem.S* is induced by tail amputation and correlates well with the progression of tail regeneration. We performed *in situ* hybridization on sections of intact and 3 dpa tadpoles, which revealed that *rfem.L-* and *rfem.S*-expressing cells were scattered around the regeneration bud and adjacent regions at 3 dpa (Fig. 1C), but were rarely observed in intact tadpole tails (Fig. 1D). We also observed scattered *rfem.L-* and *rfem.S*-expressing cells around the head and abdomen in both intact and amputated tadpoles (Fig. S1C). The number of *rfem.L-* and *rfem.S*-expressing cells at the regeneration bud and adjacent regions at 1 dpa increased significantly compared with the corresponding region in the intact tail (Fig. 1E), it is possible that the upregulation of *rfem.L* and *rfem.S* expression after tail amputation is due to the migration of *rfem.L-* and *rfem.S*-expressing cells to the amputation site or regeneration bud.

### *rfem.L* and/or *rfem.S* are required for normal tail regeneration

**Fig. 2.**
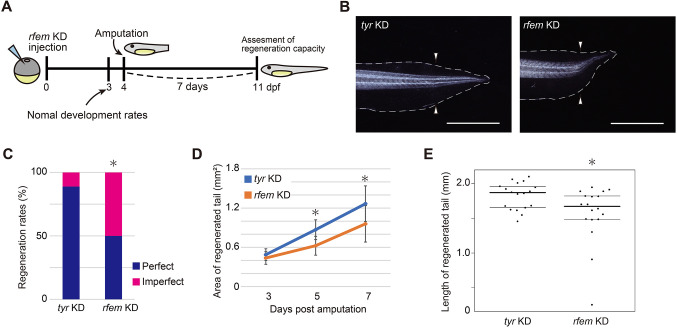
***rfem.L* and/or *rfem.S* are required for normal tail regeneration.** (A) Experimental design of *rfem.L* and *rfem.S* KD. (B) Representative images of regenerated tails at 7 dpa in tadpoles from *tyr* KD, and *rfem.L* and *rfem.S* KD groups. Gray dashed lines indicate the outline of the tail. Arrowheads indicate amputation sites. Scale bars: 2* *mm. Other examples of regenerated tails are shown in Fig. S2D. (C) Regeneration rates of *tyr* KD (*n*=18), and *rfem.L* and *rfem.S* KD (*n*=18) groups. Tadpoles from each experimental group were classified into two groups depending on the morphology of the regenerated tails: perfect or imperfect (see Materials and Methods). *P<0.05 versus *tyr* KD, Fisher's exact test. Data are representative of three experiments; the other results are shown in Fig. S2E. (D,E) Measured (D) area and (E) length of regenerated tails at (D) 3, 5 and 7 dpa, and (E) 7 dpa in tyr KD (*n*=18), and *rfem.L* and *rfem.S* KD (*n*=18) groups. Data are mean±s.d. in D. In E, the horizontal lines indicate the 25th, 50th and 75th percentiles. Other results are shown in Fig. S2F,G.*P<0.05, Welch's *t*-test.

Next, we examined the function of *rfem.L* and *rfem.S* in tail regeneration by KD using the CRISPR/Cas9 system. We injected *cas9* mRNA and guide RNAs (gRNAs) that target the coding sequence of *rfem.L* and *rfem.S* into one-cell stage embryos to introduce mutations in the *rfem.L* and *rfem.S* sequences. Tadpoles obtained by this method are mosaics of gene-edited and unedited cells because gene editing occurs in each cell independently after cleavage. We refer to the mosaic tadpoles as KD. To improve the efficiency of KD by gene editing, two gRNAs with different target sites were used for *rfem.L* and *rfem.S*, respectively (Fig. S2A). Tadpoles injected with *cas9* mRNA and gRNAs targeting *tyrosinase*, a gene responsible for albinism and unnecessary for tadpole survival, were used as controls (*tyr* KD). Injected embryos were maintained for 4 days (stage 41), and normally developed tadpoles were used for the experiments (Fig. 2A and Fig. S2B). The development rates did not differ between the *rfem.L* and *rfem.S* KD and *tyr* KD groups (Table S2). To confirm that gene editing had occurred, some of the *rfem.L* and *rfem.S* KD tadpoles were randomly selected for Inference of CRISPR Edits-Analysis (ICE analysis) (Hsiau et al., 2018 preprint), a method to estimate the proportion of cells with frameshifts or insertions/deletions ≥21 bp as a KO score. At each gRNA target site of *rfem.L* and *rfem.S* KD tadpoles, gene editions that would result in loss of function were detected (Fig. S2C). The KO scores did not differ significantly between *rfem.L* and *rfem.S* KD tadpoles with developmental abnormalities and those with normal development (Fig. S2C), suggesting that the developmental abnormalities exhibited by some *rfem.L* and *rfem.S* KD tadpoles were not due to *rfem.L* and *rfem.S* KD. Considering that *rfem.L* and *rfem.S* are rarely expressed in early development (Fig. S1A), we estimate that the effect of *rfem.L* and *rfem.S* KD on development is limited. We amputated the tails of injected tadpoles at 4 days post-fertilization (4 dpf), and the regenerative ability depending on the morphology of the regenerated tails was evaluated at 7 dpa. Several tadpoles in the *rfem.L* and *rfem.S* KD groups showed defects of tissues or regenerated tails that were bent in strange directions (Fig. 2B and Fig. S2D), and the *rfem.L* and *rfem.S* KD groups exhibited a reduced regeneration rate compared with the *tyr* KD groups (Fig. 2C and Fig. S2E, and Table S2). Measurement of the area and length of regenerated tail revealed that the *rfem.L* and *rfem.S* KD tadpoles regenerated significantly smaller and shorter tails (Fig. 2D,E and Fig. S2F-H). These results indicated that *rfem.L* and/or *rfem.S* are required for normal tail regeneration. The morphology of regenerated tails of *rfem.L* and *rfem.S* KD tadpoles remained abnormal as they grew (Fig. S2J) and the tadpoles showed poor regeneration outcomes after re-amputation (Fig. S2K).

### *rfem.L* and *rfem.S* are expressed in a subset of leukocytes

**Fig. 3.**
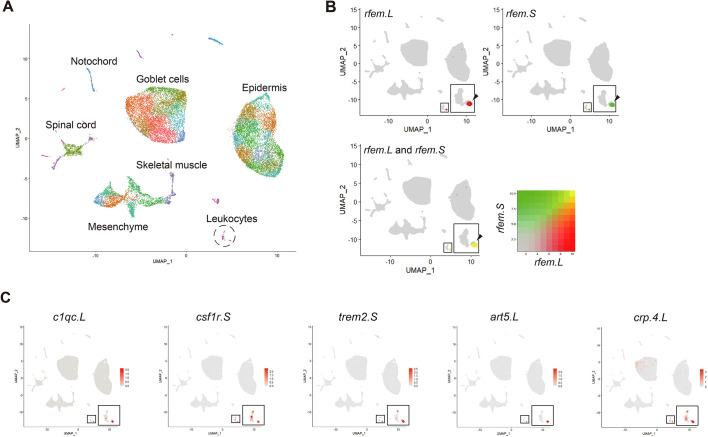
***rfem.L* and *rfem.S* are expressed in a subset of leukocytes with a macrophage-like gene expression profile.** (A) UMAP plot of cells detected in the scRNA-seq. Cells from the tail tips and tail stumps from intact tadpoles, and the regeneration bud at 2 dpa in tadpoles are plotted together and divided into 50 clusters shown in different colors. (B) *rfem.L* and *rfem.S* expression on a UMAP plot. (Upper left) *rfem.L* and (upper right) *rfem.S* expression are plotted using red and green dots, respectively. (Lower) Expression of *rfem.L* and *rfem.S* is shown by merged colors. Insets in each plot are magnified images of the leukocyte clusters, which are outlined in the plots. Black arrowheads indicate the fraction of the *rfem.L-* and *rfem.S*-expressing cells. (C) Genes with enriched expression in the *rfem.L-* and *rfem.S-*expressing cells.

To estimate the function of *rfem.L* and *rfem.S*, we performed RNA-seq of tail stumps of amputated *rfem.L* and *rfem.S* KD tadpoles. The RNA-seq detected six genes that were significantly downregulated in *rfem.L* and *rfem.S* KD tail stumps (Table S3), including three hemoglobin genes, suggesting that *rfem.L* and *rfem.S* KD affects wound repair, including blood vessel formation. We also attempted to identify the cell types that express *rfem.L* and *rfem.S* using scRNA-seq on enzymatically dispersed intact tails and 2 dpa regeneration buds (Fig. S3A). Cell clusters that reflect most of the tissues constituting the tail, such as the epidermis, muscles, notochord, nerves and blood cells, were detected (Fig. 3A and Fig. S3B,C), suggesting that the data covered a broad range of tissues in the tail. *rfem.L* and *rfem*.S were expressed in a few cells, and their expression was well correlated (Fig. 3B). The *rfem.L-* and *rfem.S-*expressing cell fraction was found in both intact tails and regeneration buds (Fig. S3D), and the fraction belonged to a cluster of leukocytes, indicating that *rfem.L-* and *rfem.S*-expressing cells are a type of leukocyte. We examined genes that are characteristically expressed in the fraction and found that expression of *c1qc.L*, *csf1r.S*, *trem2.S*, *art5.L* and *crp.4.L* was enriched (Fig. 3C). In particular, the expression of *c1qc.L* and *art5.L* correlated well with that of *rfem.L* and *rfem.S*, and these two genes were identified in the first screening of this study (Table S1). Complement component 1 subcomponent C chain (C1q) is produced in tissue phagocytes such as macrophages and dendritic cells (Thi et al., 2017). *colony stimulating factor-1 receptor* (*csf1r*) and *triggering receptor expressed on myeloid cells 2* (*trem2*) are known to be expressed on myeloid cells such as macrophages and dendritic cells (Stanley and Chitu, 2014; MacDonald et al., 2005; Hickman and El, 2014). These results revealed that *rfem.L-* and *rfem.S*-expressing cells had a macrophage-like gene expression profile.

Because *rfem.L* and *rfem.S* are identified as genes that are downregulated in *il11* KD tadpoles (Fig. S3E), we assessed whether the *rfem.L-* and *rfem.S*-expressing cells respond to Il11 by analyzing their *il11 receptor subunit alpha* (*il11ra.L*) expression, and found that *il11ra.L* expression was not detected (Fig. S3F). Therefore, it is possible that *rfem.L-* and *rfem.S*-expressing cells do not receive Il11 directly and thus are regulated indirectly by Il11 signaling. We could not, however, exclude the possibility that the *rfem.L-* and *rfem.S*-expressing cells express *il11ra.L* under the detection threshold because of the relatively low sensitivity of detection of gene expression by scRNA-seq.

### Expression of *rfem.L* or *rfem.S* in macrophage-like cells is required for tail regeneration

**Fig. 4.**
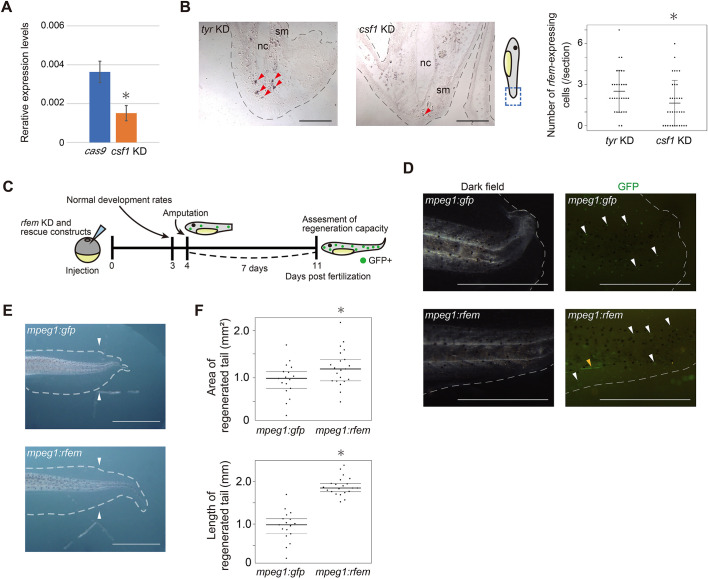
***rfem.L* or *rfem.S* expression in macrophage-like cells is required for tail regeneration.** (A) Relative expression levels of *rfem.L* and *rfem.S* in the tail stumps at 24 h post-amputation in tadpoles from *cas9* and *csf1* KD groups. Vertical axes represent relative expression levels normalized to those of *ef1a*. Data are mean±s.d., *n*=3. *P<0.05, Welch's *t*-test. (B) (Left) Representative images of *in situ* hybridization of *rfem.L* and *rfem.S* on sagittal sections of tails from 1 dpa *tyr* KD and *csf1* KD tadpoles. Red arrowheads indicate signals. Blue dashed lines in the schematic diagrams outline the location of the sections. nc, notochord; sm, skeletal muscle. Scale bars: 200 μm. (Right) Number of *rfem.L-* and *rfem.S*-expressing cells in sections of 1 dpa tails of *tyr* KD (31 sections from seven tadpoles) or *csf1* KD (34 sections from eight tadpoles) tadpoles. Data are mean±s.d., *P<0.05, Welch's *t*-test. (C) Design of *rfem.L* and *rfem.S* rescue experiment. Normal development ratios are shown in Table S5. (D) Representative images of GFP-expressing cells in regenerated tails at 7 dpa in *rfem.L* and *rfem.S* KD tadpoles co-injected with the *mpeg1:gfp* (control) or *mpeg1:rfem* (*rfem*-rescued) constructs. Gray dashed lines indicate the outline of the tail. White arrowheads indicate representative GFP-expressing cells. The yellow arrowhead indicates ectopic expression of GFP in muscle. Scale bars: 1* *mm. (E) Representative image of regenerated tails at 7 dpa in a control, and an *rfem.L*-rescued *rfem* KD tadpole. Gray dashed lines show the outline of the tail. Arrowheads indicate amputation sites. Scale bars: 2* *mm. (F) Measured area and length of regenerated tails at 7 dpa in control (*n*=17) and *rfem.L*-rescued (*n*=21) groups. *P<0.05, Welch's *t*-test. Data are representative of three experiments; the other results are shown in Fig. S4K,L.

To assess the relationship between tail regenerative capacity, and *rfem.L* and *rfem.S* expression in leukocytes, we examined the correlation between the area of the regenerated tail and the KO score in the peripheral blood cell (PBC) fraction of *rfem.L* and *rfem.S* KD tadpoles, but failed to detect a tendency for the regenerated area to be smaller in tadpoles with higher KO scores in the fraction (Fig. S2I), possibly because of the small proportion of *rfem.L-* and *rfem.S-*expressing leukocytes in PBCs. To investigate the role of the *rfem.L-* and *rfem.S*-expressing leukocytes in tail regeneration directly, we attempted to examine the effects of depletion of *rfem.L-* and *rfem.S*-expressing leukocytes on tail regeneration. The major fraction of *rfem.L-* and *rfem.S*-expressing cells co-expressed *csf1r.S* (Fig. 3C and Fig. S4A), a receptor for *colony stimulating factor 1* (*csf1*)*.* Csf1 is a cytokine that is essential for differentiation and survival of monocytes and macrophage lineage cells in mammals (Nakamichi et al., 2013), and Csf1 is also reported to function in *X. laevis* (Grayfer and Robert, 2013). We therefore assumed that *csf1* KD depletes Csf1-dependent cells, including the *rfem.L-* and *rfem.S*-expressing leukocytes. *X. laevis* has only one *csf1* gene on the genome (*csf1.S*) and we designed two gRNAs (gRNA#4 and gRNA#5; Fig. S4B) for *csf1.S* and generated *csf1* KD tadpoles using the CRISPR/Cas9 system. Tadpoles injected with only *cas9* mRNA were used as controls (*cas9* groups). ICE analysis confirmed that gene editing was successful at the target site of each gRNA (Fig. S4C). *csf1* KD did not decrease normal development rates (Table. S4). We quantified the *rfem.L* and *rfem.S* expression levels (Fig. 4A), and the number of *rfem.L-* and *rfem.S*-expressing cells (Fig. 4B) in the tail stumps of *csf1* KD tadpoles, and found that both were significantly decreased, suggesting that *csf1* KD depleted the *rfem.L-* and *rfem.S*-expressing leukocytes. We also quantified genes with expression patterns similar to that of *rfem.L* and *rfem.S* in the scRNA-seq; expression levels of *c1qa.L*, *c1qb.L*, *c1qc.L*, *art5.L* and *crp.4.L* (Fig. S4D, E); and the number of *c1qa.L*-expressing cells (Fig. S4F). We and observed a significant reduction or a trend towards reduction of the expression levels and number in the tail stumps in *csf1* KD tadpoles. The lack of a statistically significant reduction in the cell number may be due to the difficulty in detecting *clqa.L*-expressing cells because of their low expression level. Several *csf1* KD tadpoles showed tail regeneration defects with the tails becoming thinner and bending in strange directions (Fig. S4G), and the regeneration rates of *csf1* KD groups were lower or tended to be lower than those of the control groups (Fig. S4H and Table S4).

To confirm that *rfem.L* and/or *rfem.S* expression in leukocytes is required for regeneration, we performed cell type-specific rescue experiments using the zebrafish *mpeg1* promoter, which is reported to work in *X. laevis* macrophage-like cells (Paredes et al., 2015). We designed constructs that express *rfem.L* and *acgfp1* (*Aequorea coerulescens* gene encoding green fluorescent protein) bicistronically, or only *acgfp1* under control of the *mpeg1* promoter (*mpeg1:rfem* and *mpeg1:gfp* respectively, Fig. S4I) to perform macrophage-like cell-specific rescue in *rfem.L* and *rfem.S* KD tadpoles (Fig. 4C). Scattered *gfp-*expressing cells were observed in regenerating tails of tadpoles injected with the construct (Fig. 4D and Fig. S4J), suggesting that the construct worked as expected. Compared with control tadpoles, the area and length of the regenerated tails in the *mpeg1:rfem*-rescued 7 dpa *rfem.L* and *rfem.S* KD tadpoles were significantly recovered (Fig. 4E,F and Fig. S4K,L), indicating that *rfem.L* or *rfem.S* expression in macrophage-like cells has an indispensable role in successful tail regeneration.

In this study, we identified *rfem.L* and *rfem.S* as genes required for tail regeneration, and indicated that *rfem.L* and *rfem.S* have an essential role in the regeneration-promoting function of macrophage-like cells. Leukocytes are recruited to the wound site to contribute to wound healing by killing pathogens and producing various cytokines (Koh and DiPietro, 2011; Wynn and Vannella, 2016). On the other hand, an excessive inflammatory response can lead to regenerative failure. In *X. laevis*, it is suggested that there are inflammatory and reparative myeloid cells that impair and promote tail regeneration, respectively (Aztekin et al., 2020). The *rfem.L-* and *rfem.S*-expressing macrophage-like cells identified by this study define a (sub)population of these reparative myeloid populations. We propose that *rfem.L* and *rfem.S* provide a clue to unveiling the cellular and molecular mechanisms that promote regenerative ability in leukocytes.

## MATERIALS AND METHODS

### Animals

*X. laevis* adults were purchased from domestic breeders (Hamamatsu Seibutsu Kyouzai, Shizuoka, Japan; Watanabe Zoushoku, Hyogo, Japan). Tadpoles were obtained by mating *X. laevis* adults or by artificial fertilization. Adult frogs and tadpoles were kept at 20°C. Surgical procedures such as dissections and amputations were performed under 0.02% ethyl 3-aminobenzoate methanesulfonate salt (MS-222, Millipore Sigma) or ice anesthesia. Tail amputation was performed at 4 days post-fertilization (4 dpf; stage 41). Tadpoles were randomly assigned to experimental groups. Animals used in this study were grown under identical conditions without blinding. Animal experiments were performed in accordance with the Guidelines for Proper Conduct of Animal Experiments of Science Council of Japan. The protocols of experiments using living modified organisms in this study were approved by the Committee on Living Modified Organism Experiments of the Graduate School of Science at the University of Tokyo (DNA Exp 17-3).

### Sample size

No statistical methods were used to predetermine the sample size.

### Quantitative reverse transcription -PCR (qRT-PCR)

To quantify the *rfem.L-* and *rfem.S* expression in the tail stumps after tail amputation, we amputated tadpole tails at 4 dpf and sampled four batches of 20 tail stumps at 0, 1, 3, 5 and 7 dpa. Total RNA was extracted using an RNeasy Mini Kit (Qiagen) and Micro Smash MS-100 (Tomy Seiko). cDNAs were synthesized from the same amounts of total RNAs per sample using a PrimeScript RT reagent Kit with gDNA Eraser (Perfect Real Time) (Takara). A group without reverse transcription (RT-) was also prepared as a control. Real-time-PCR was performed with SYBR premix ExTaq II (Takara) or TB Green Ex Taq II (Tli RNaseH Plus) (Takara) and LightCycler 480 (Roche). Sequences of primers used are shown in Table S6. Because the sequences of *rfem.L* and *rfem.S* are quite similar and it was difficult to design primers specific to *rfem.L* or *rfem.S*, respectively, and the amplicon sizes are within 80-150 bp for qRT-PCR, we designed primers for the consensus sequences of *rfem.L* and *rfem.S* to amplify both of them. The threshold cycle was calculated using the 2nd derivative maximum method. The relative expression levels were normalized to those of *elongation factor 1 alpha.* We electrophoresed the PCR products and confirmed that no amplification was detected in the RT− group. Tukey-Kramer's test was performed using R (v3.6.3).

To quantify the gene expression in *csf1* KD tadpoles, we amputated the tails of *csf1* KD and *cas9* tadpoles, and sampled three batches of 12 tail stumps at 1 day post amputation, and qRT-PCR was performed as described above.

### *In situ* hybridization

For cloning the *rfem.L* and *rfem.S* partial sequences, cDNA was synthesized from the total RNA of thymi of *X. laevis* J strain stage 52 tadpoles with TRIzol reagent (Thermo Fisher Scientific) and SuperScript III Reverse Transcriptase (Thermo Fisher Scientific); then, the partial sequences of *rfem.L* and *rfem.S* were amplified by PCR with the cDNA, and the PCR products were cloned into a pGEM-T Easy vector (Promega). Sequences of the primers used are shown in Table S6. To synthesis RNA probes, the cloned vectors were amplified by PCR, and then PCR products were purified using FastGene Gel/PCR Extraction Kit (Nippon Genetics). Digoxigenin (DIG)-labeled RNA probes were synthesized using DIG RNA labeling Mix (Roche) and T7 or SP6 RNA polymerase (Roche) with the purified PCR products as templates. The probes were purified by ethanol precipitation. We synthesized probes for *rfem.L* and *rfem.S*, and the synthesis was confirmed by denaturing gel electrophoresis.

*In situ* hybridization was performed as described previously (Tsujioka et al., 2017; Hatta-Kobayashi et al., 2016) with several modifications. Intact 4 dpf tadpoles, and tadpoles amputated at 4 dpf at 3 dpa were fixed in MEMFA fixative [0.1 M MOPS (pH 7.4), 2 mM EGTA (pH 8.0), 1 mM MgSO_4_ and 10% formalin) at room temperature for 48 h. They were dehydrated in an ethanol and Clear Plus (Falma) series and embedded in Paraplast plus (Leica); 8 µm sections were prepared and adhered to MAS-coated slide glass (Matsunami Glass). Sections were deparaffinized and rehydrated with a Clear Plus and ethanol series, permeabilized with 20 µg/ml proteinase K (Roche) and 1% Triton X-100, followed by acetylation with 0.25% acetic anhydride. The mixture of DIG-labeled *rfem.L* and *rfem.S* probes was hybridized in buffer containing 50% formamide, 5× SSC (75 mM trisodium citrate and 750 mM NaCl), 240 µg/ml torula yeast RNA (Millipore Sigma), 500 µg/ml salmon sperm DNA (FUJIFILM Wako Chemical) and 50× Denhardt's solution (FUJIFILM Wako) at 58°C for 16 h. Slides were washed with 0.2× SSC at 58°C. Sections were incubated with 1/5000 diluted alkaline phosphatase-conjugated anti-DIG antibody (catalogue code 11175041910, lot 44053100, Roche). Signals were detected with NTM buffer [100 mM Tris (pH 9.5), 100 mM NaCl and 50 mM MgCl_2_] supplemented with 1/100 volume of NBT/BCIP stock solution (Roche). The endogenous melanin pigments were bleached by placing the slides in 0.5× SSC containing 0.3% H_2_O_2_ under fluorescent light overnight. The sections were mounted with 50% glycerol. The slides were observed using a differential interference microscope.

### Knockdown experiments

KD experiments using the CRISPR/Cas9 system were performed essentially as described previously (Tsujioka et al., 2017; Kato et al., 2021). Guide RNAs were synthesized as follows. The *rfem.L*, *rfem.S, tyr.L* and *tyr.S* sequences were obtained from Xenbase (RRID:SCR_003280; Xetrov90002578*m*.L for *rfem.L* and Xetrov90002579*m*.S for *rfem.S*, respectively). The *csf1.S* sequence (|JX418294.1) (Grayfer and Robert, 2013) was obtained from the NCBI (https://www.ncbi.nlm.nih.gov/). The guide RNAs were designed with CRISPRdirect (http://crispr.dbcls.jp/) (Naito et al., 2015). Two guide RNAs per gene were designed to improve the KD efficiency. The target sequences are shown in Table S6. The guide RNA #1 targets the same sequences of *rfem.L* and *rfem.S* (Fig. S2A). The target sequences were inserted into the DR274 plasmid (Hwang et al., 2013) (for gRNAs #1, #2 and #3) or the DR274-T7dG1 plasmid [for gRNAs #4 and #5; modified DR274 plasmid was used for synthesis of gRNAs that have one guanine at the 5′ end; DR274 was digested with *Bsa*I (New England Biolabs) and the GAGACCGAGAGAGGGTCTCA sequence was inserted downstream of the T7 promoter to generate the DR274-T7dG1], and guide RNA was synthesized using an AmpliScribe T7-Flash Transcription Kit (Lucigen) then purified with an RNeasy Mini Kit. The synthesis was confirmed by denaturing gel electrophoresis.

For *cas9* mRNA synthesis, pXT7-hcas9 (China Zebrafish Resource Center) (Chang et al., 2013) was digested with *Xba* I (Takara) and then mRNA was synthesized using mMESSAGE mMACHINE T7 ULTRA Kit (Thermo Fisher Scientific) and purified with an RNeasy Mini Kit. The synthesis was confirmed by denaturing gel electrophoresis.

Artificial fertilization was performed as follows. Unfertilized eggs were obtained by squeezing an adult female frog that was injected with 500 units of gonadotropin (ASKA Pharmaceutical) the previous night. A sperm suspension was prepared by shredding a part of a testis in 0.33× De Boer solution (18.3 mM NaCl, 217 μM KCl, 73 μM CaCl_2_ and 695 μM NaHCO_3_). Eggs were fertilized by mixing with sperm suspension. Fertilized eggs were dejellied with 3% cysteine (pH 7.6) and chilled to 12°C in 0.1×MMR [10 mM NaCl, 200 µM KCl, 100 µM MgSO_4_, 200 µM CaCl_2_, 500 µM HEPES (pH 7.4)].

We injected 18.4 nl of the guide RNA and *cas9* mRNA solution into the animal hemisphere of healthy one-cell stage embryos in 0.1×MMR supplemented with 2% Ficoll PM 400 (Cytiva) at 12°C using a Nanoject II (Drummond Scientific Company). Concentrations of *cas9* mRNA and guide RNA were as follows: guide RNA, 80 ng/µl each; *cas9* mRNA, 700 ng/µl for *rfem.L* and *rfem.S* KD, and 1000 ng/µl for *csf1* KD. Embryos of control group were injected with guide RNA#6 and #7 (*tyr* KD) and *cas9* mRNA at the same concentration as the experimental group. Injected embryos were kept separately in 96-well plates (one embryo/well) in 0.1× MMR at 12°C for 24 h and then reared at 20°C.

Rates of normally developed tadpoles were counted at 3 dpf (stage 36-39), then healthy tadpoles with a normal morphology and motility were transferred to 0.1× Steinberg solution [5.8 mM NaCl, 67 µM KCl, 34 µM Ca(NO_3_)_2_, 83 µM MgSO_4_ and 300 µM HEPES (pH7.4)] in a 90 mm dish. We used any mRNA and gRNA-injected, surviving and healthy tadpoles at 4 dpa for each experiment. Tadpole tails were amputated at 4 dpf, maintained for 7 days and then classified into two groups depending on the morphology of the regenerated tails, as follows: perfect, regenerated tail with normal morphology of muscle, notochord and fin; imperfect, no tail regeneration or regenerated tail with defects in any tissues and/or vent axis. Dead tadpoles or tadpoles with severe malformation of the whole body were excluded from counting. Statistical significance of the ratio of perfect/imperfect was assessed by two-sided Fisher's exact test using R (v3.6.3). Measurement of the regenerated tails were performed as described previously (Suzuki et al., 2022); we took photos of the regenerated tails of tadpoles and the parameters (area and length of regenerated tail) were measured using Fiji (Schindelin et al., 2012).

### Estimation of KO scores of gRNA target sites

To estimate the KO scores, genomic DNA was extracted from the whole body, amputated tail or blood cells (sample types depend on each experiment) by boiling them in 50 mM NaOH at 98°C for 10 min, followed by neutralization with 1/10 volume of 1 M Tris-HCl (pH 7.5).

PBCs were collected as described previously (Naora et al., 2013) with minor modifications. After tail amputation, each tadpole was immersed in 0.6% sodium chloride with 2.5 mM EDTA in a 0.2 ml PCR tube and left to bleed for 45 min. The tadpoles were then removed and the resulting blood fraction was centrifuged at 500 ***g*** for 5 min to pellet blood cells. The supernatant was removed and pelleted blood cells were resuspended in 10 µl of 50 mM NaOH, and genomic DNA was extracted as described above.

PCR and Sanger sequencing were performed with the primers listed in Table S6. Sequence data were subjected to ICE analysis (Hsiau et al., 2018 preprint) (https://ice.synthego.com/) to estimate the KO scores (proportion of cells with either a frameshift, or at least a 21 bp insertion/deletion.).

### Single-cell RNA sequencing (scRNA-seq)

Tails of intact tadpoles at 4 dpf and tadpoles at 2 dpa whose tails were amputated at 4 dpf (as ‘regeneration bud’) were subjected to scRNA-seq. Intact tails were divided into anterior (as ‘tail stump’) and posterior (as ‘tail tip’) parts (Fig. S3A). Tails were shredded using a razor. Cells were then enzymatically dispersed by treating them in 1× PBS (137 mM NaCl, 2.7 mM KCl, 10 mM Na_2_HPO_4_ and 1.8 mM KH_2_PO_4_) supplemented with 0.4 mg/ml of Liberase TH Research Grade (Roche) and 177.7 units/μl DNase I (Thermo Fisher Scientific) for 40 min on a rotator at 30°C. Dispersed cells were washed and suspended in 1× sorting buffer [1× PBS supplemented with 1 mM EDTA, 25 mM HEPES and 1% BSA (Nacalai Tesque)], then filtered through a 55 μm opening nylon mesh (AS ONE, Osaka, Japan). Dead cells were stained by adding 1/100 volume of 7-AAD (TONBO Biosciences). Live cells in the cell suspension (7-AAD negative) were sorted using a FACS Aria III (Becton, Dickinson and Company). Doublet or multiplet events were gated out using height and width parameters of forward and side scatter. Sorted cells were used to generate scRNA-seq libraries using Chromium Single Cell 3′ Reagent Kits v3 (10X Genomics), followed by sequencing on a Novaseq 6000 (Illumina).

Output data were processed using Cell Ranger v3.0.2 to generate a gene count matrix. We used a genome sequence file generated by combining the *X. laevis* genome version 9.1 sequence (Xla.v91.repeatMasked.fa) and mitochondrial genome sequence of genome version 9.2 (XL9_2.fa) for mapping, and a gene model file generated by combining the gene model for genome version 9.1 (XL_9.1_v1.8.3.2.primaryTranscripts.gff3) and mitochondrial gene annotation of the gene model for genome version 9.2 (XENLA_9.2_Xenbase.gff3) for read counting. The genome sequence files and the gene model files were obtained from Xenbase. The generated count matrix was analyzed using Seurat package v3.2.2 (Stuart et al., 2019). The cells whose percentage of mitochondrial gene counts per total gene counts exceeded 20, or whose total gene counts were outside a 500-10,000 range were excluded from the analysis. The numbers of cells used for analysis were as follows: tail stump, 6622; tail tip, 7600; regeneration bud, 7400. The gene counts were normalized by the total expression of each cell and scaled; linear dimensional reduction was then performed by principal component analysis. For clustering the cells, we used FindNeighbors and FindClusters functions on Seurat using PC1-PC100, which divided cells to 50 clusters, and processed data was visualized using Uniform Manifold Approximation and Projection (UMAP) (Mcinnes et al., 2018 preprint).

### Analysis of the published RNA-seq data

The RNA-seq data of the *X. laevis* embryo developmental expression (Session et al., 2016) and tail stumps at 48 h after amputation of *il11* KD and control tadpoles (Tsujioka et al., 2017) were analyzed using HISAT2 v2.1.0 (Kim et al., 2019) for mapping, and HTSeq v0.11.2 (Anders et al., 2015) for read counting, with the genome sequence file (Xla.v91.repeatMasked.fa) and the gene model file (XL_9.1_v1.8.3.2.primaryTranscripts.gff3). Differential expression analysis was performed using DESeq2 package v1.32.0 (Love et al., 2014).

### RNA-seq of tail stumps of *rfem* KD tadpoles

*rfem.L* and *rfem.S* KD tadpoles were obtained as described above. For the control group, tadpoles injected with only *cas9* mRNA were used. We amputated the tails of tadpoles at 4 dpf, then 13-20 tail stumps of tadpoles were sampled 48 h after amputation. We collected samples from three different batches. The samples were homogenized using TRIzol reagent (Thermo Fisher Scientific), and extraction of total RNA and the following RNA-seq were performed at Tsukuba i-Laboratory LLP (Ibaraki, Japan). Extracted total RNA was subjected to mRNA isolation using NEBNext Poly(A) mRNA Magnetic Isolation Module (New England Biolabs). cDNA libraries were produced using a NEBNext Ultra Directional RNA Library Prep Kit for Illumina (New England Biolabs). Sequencing was performed using a NextSeq500 (Illumina) to generate ∼4×10^7^ paired-ends reads (36 bp ×2) from each cDNA library. The RNA-seq data were analyzed as described above.

### Cell type-specific rescue experiments

The rescue construct was generated as follows. Two I-SceI meganuclease recognition sites were inserted between *Sph*I and *Pst*I sites, and *Kpn*I and *Sac*I sites, respectively, of the pUC19 plasmid to generate the pIS2 plasmid. A DNA fragment containing the zebrafish *mpeg1* promoter sequence (Paredes et al., 2015), *rfem.L*-coding sequence, P2A, *acgfp1*-coding sequence, and SV40 polyadenylation signals were synthesized by Twist Bioscience. Synonymous substitutions were introduced into the *rfem.L* sequence at gRNA#1 and #2 target sites [#1, GGGATtTAttctTCtATcCC; #2, GGAGCcTGGGTttTaTAcCA (substitutions are indicated with a lowercase letter)]. A DNA fragment without the *rfem.L*-coding sequence and P2A was also synthesized for the control experiment. pIS2 plasmid was digested with XbaI and the DNA fragment was inserted using an In-Fusion HD cloning kit (Takara) to generate *mpeg1:rfem* and *mpeg1:gfp* constructs, respectively.

Cell type-specific *rfem.L* forced-expression in *rfem.L* and *rfem.S* KD tadpoles was performed as follows. The constructs had two I-SceI sites to perform I*-*SceI mediated transgenesis, which would facilitate the expression by stably inserting into the genome, in addition to the free constructs remaining in the cells of the tadpole at the time of tail amputation. I-SceI mediated transgenesis was performed as described previously (Ogino et al., 2006) with several modifications. We prepared a solution containing 1.25 µM of Cas9 protein (PNA Bio), 13.3 ng/µl of *rfem.L/S* gRNA #1, #2 and #3, respectively, 0.5 units/µl of I-SceI (New England Biolabs), 75 ng/µl of *mpeg1:rfem* or *mpeg1:gfp* constructs, and 1×CutSmart Buffer (New England Biolabs). The solution was incubated at 37°C for 30 min, then 18.4 nl was injected into one-cell stage embryos as described above. Injected embryos were maintained at 20°C. Tails of tadpoles were amputated at 4 dpf, followed by assessment of the regenerative capacity by measuring of the area and length of the regenerated tails at 7 dpa. A two-sided Fisher's exact text was performed using R (v3.6.3)

### General statistical analyses

All statistical analyses were conducted in R (v3.6.3). The statistical method and sample size are provided in the corresponding figure legends.

## Supplementary Material

10.1242/develop.200467_sup1 Supplementary information
