# Supplementary material for: regeneration factors expressed on myeloid expression in macrophage-like cells is required for tail regeneration in Xenopus laevis tadpoles
Source: Development. 2023 Jul 31;150(15):dev200467. doi: 10.1242/dev.200467 (PMC10445729; doi:10.1242/dev.200467)
Supplement: Supplementary information [file develop-150-200467-s1.pdf]

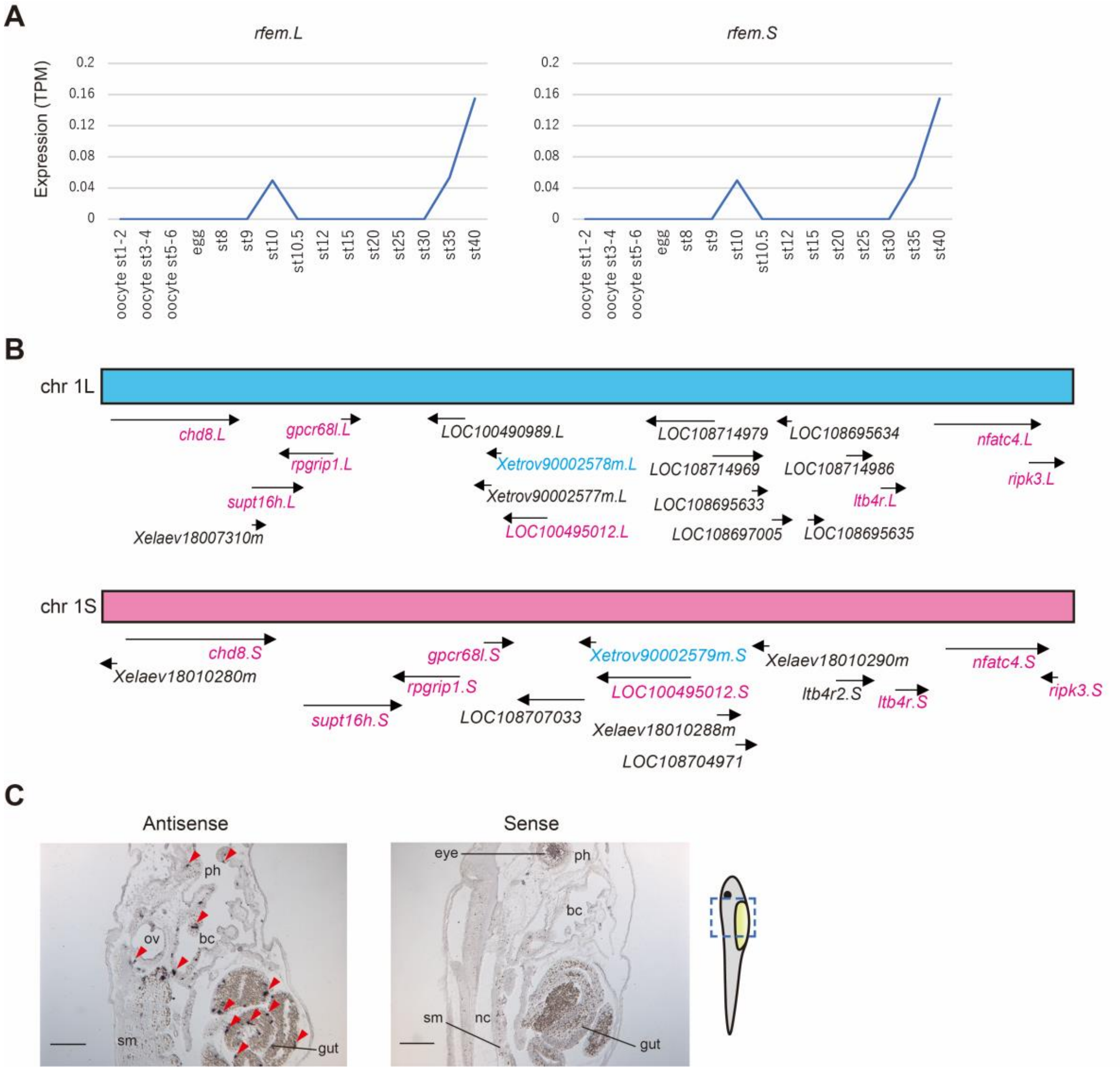

Fig. S1. Analysis of developmental and site-specific expression pattern, and the synteny of *rfem.L/S*. (A) Expression level of *rfem.L* and *rfem.S* in the RNA-seq of the early development period reported by Session *et al.*[S1]. TPM, transcripts per million reads. (B) Structures of the neighboring regions of *rfem.L* (*Xetrov90002578m.L*) and *rfem.S* (*Xetrov90002579m.S*) on chromosome 1L and 1S are shown. Genes whose positions are conserved in the L and S chromosomes are indicated in magenta. (C) Representative images of *in situ* hybridization of *rfem.L/S* on sagittal sections of the anterior region. Red arrowheads indicate representative signals of *rfem.L/S* expression. Box with blue dashed lines in the schematic diagrams indicate the location of the section. nc, notochord; sm, skeletal muscle; ov, otic vesicle; ph, pharynx; bc, branchial cavity. Scale bars: 200 µm. Antisense, 8 sections from 6 individuals; sense, 8 sections from 3 individuals.

Development • Supplementary information

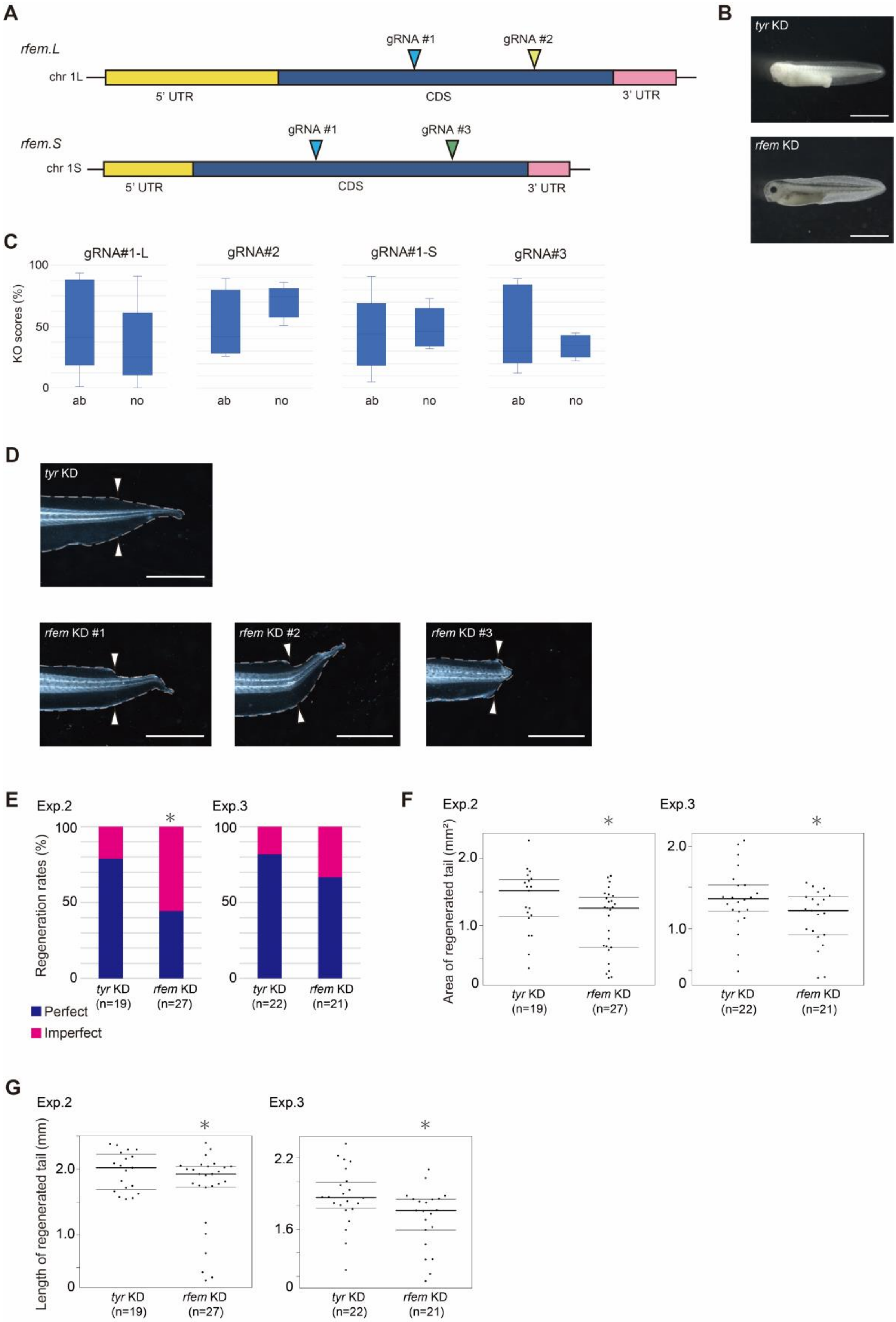
A
rfem.L
chr 1L
gRNA #1
gRNA #2
5' UTR
CDS
3' UTR
rfem.S
chr 1S
gRNA #1
gRNA #3
5' UTR
CDS
3' UTR
B
tyr KD
rfem KD
C
gRNA#1-L
gRNA#2
gRNA#1-S
gRNA#3
KO scores (%)
100
50
0
ab
no
D
tyr KD
rfem KD #1
rfem KD #2
rfem KD #3
E
Exp.2
Exp.3
Regeneration rates (%)
100
50
0
tyr KD (n=19)
rfem KD (n=27)
tyr KD (n=22)
rfem KD (n=21)
Perfect
Imperfect
F
Exp.2
Exp.3
Area of regenerated tail (mm²)
2.0
1.0
0
tyr KD (n=19)
rfem KD (n=27)
tyr KD (n=22)
rfem KD (n=21)
G
Exp.2
Exp.3
Length of regenerated tail (mm)
2.0
1.0
0
2.2
1.6
tyr KD (n=19)
rfem KD (n=27)
tyr KD (n=22)
rfem KD (n=21)

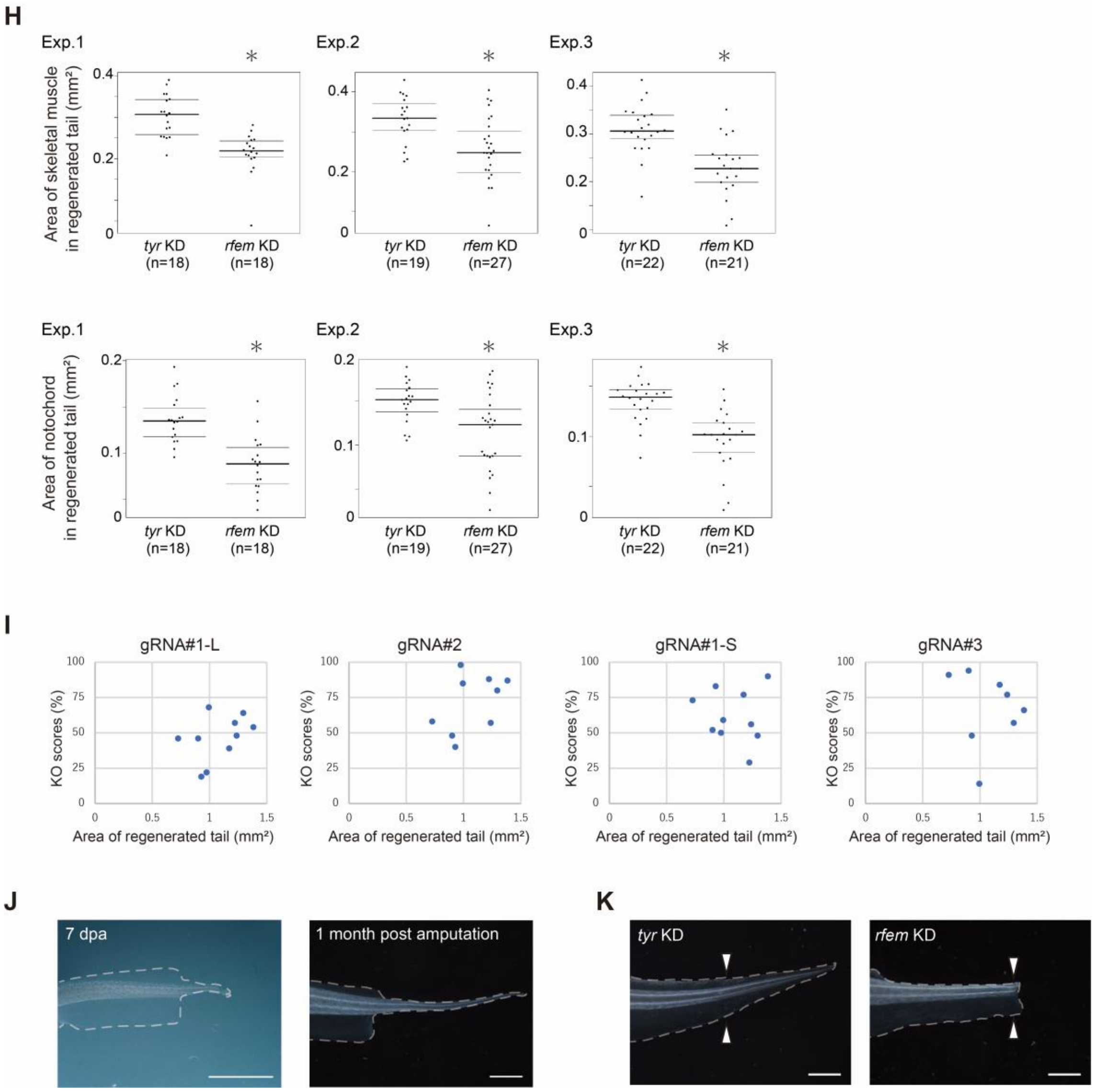

Fig. S2. *rfem* is not essential for development, but is required for normal tail regeneration.

(A) Target sites of guide RNAs used for KD of *rfem* are indicated by the arrowheads. gRNA#1 targets common sequences in the *rfem.L* and *rfem.S* genes. There are no introns within the *rfem.L/S* genes. (B) Representative images of normally developed 4 dpf tadpoles from the *tyr* KD and *rfem* KD groups. Scale bars: 2 mm. (C) Box plots of KO scores for the gRNA #1, #2, and #3 target sites in *rfem.L* and *rfem.S* estimated using genomic DNA extracted from the whole body of each *rfem* KD tadpole with abnormal morphology (ab, *n*=5) or normal morphology (no, *n*=5) at 3 days after fertilization. No significant differences were detected using Welch's t test. (D) Other examples of abnormal tail regeneration observed in the *rfem* KD group. Gray dashed line shows the outline of the tail. Arrowheads indicate amputation site. Scale bars: 2 mm. (E) Regeneration rates of the *tyr* KD and *rfem* KD groups.

Development • Supplementary information

Tadpoles from each experimental group were classified into 2 groups depending on the morphology of the regenerated tails: perfect or imperfect (see Methods). Three experiments, including those for which the results are shown in Fig. 2C, were performed independently. The numbers of individuals in each experimental group are indicated below each graph. *$P < 0.05$ versus *tyr* KD, Fisher's exact test. (F, G) Measured (F) area and (G) length of regenerated tails at 7 dpa. Three experiments, including those for which the results are shown in Fig. 2D and 2E, were performed independently. The horizontal lines indicate the 25th, 50th, and 75th percentiles.*$P < 0.05$, Welch's t test. (H) Measured area of skeletal muscle and notochord in regenerated tail from tadpoles in the *tyr* KD and *rfem* KD groups at 7 days after amputation. The data for the first experiment are from the experiment shown in Fig. 2D. The horizontal lines indicate the 25th, 50th, and 75th percentiles.*$P < 0.05$, Welch's t test. (I) Scatter plots of measured area of regenerated tail at 7 days after amputation (horizontal axis) and KO scores at each gRNA target site estimated using genomic DNA extracted from blood cells (vertical axis) of *rfem* KD tadpoles. gRNA#1-L and gRNA#1-S represent the gRNA #1 target site in *rfem.L* and *rfem.S*, respectively. *n*=8-10. No correlation was detected between the 2 parameters. (J) Representative images of *rfem* KD tadpoles showing (left) imperfect regeneration at 7 days after amputation, and (right) the same individual at 1 month after amputation. We traced the regeneration outcomes of 3 *rfem* KD tadpoles showing imperfect regeneration for 1 to 2 months, and observed that the morphology remained imperfect in all of them. Gray dashed line shows the outline of the tail. Scale bars: 2 mm. (K) Representative images of regenerated tail at 7 days after the second amputation in tadpoles that had been amputated twice (at 4 days after fertilization and at 1 to 2 months after the first amputation). We performed this experiment using 3 *rfem* KD tadpoles showing imperfect regeneration after the first amputation and 7 *tyr* KD tadpoles, and observed that the 3 *rfem* KD tadpoles also showed imperfect regeneration after the second amputation, in contrast to the result of the 6 *tyr* KD tadpoles which showed perfect regeneration. Scale bars: 2 mm.

Development • Supplementary information

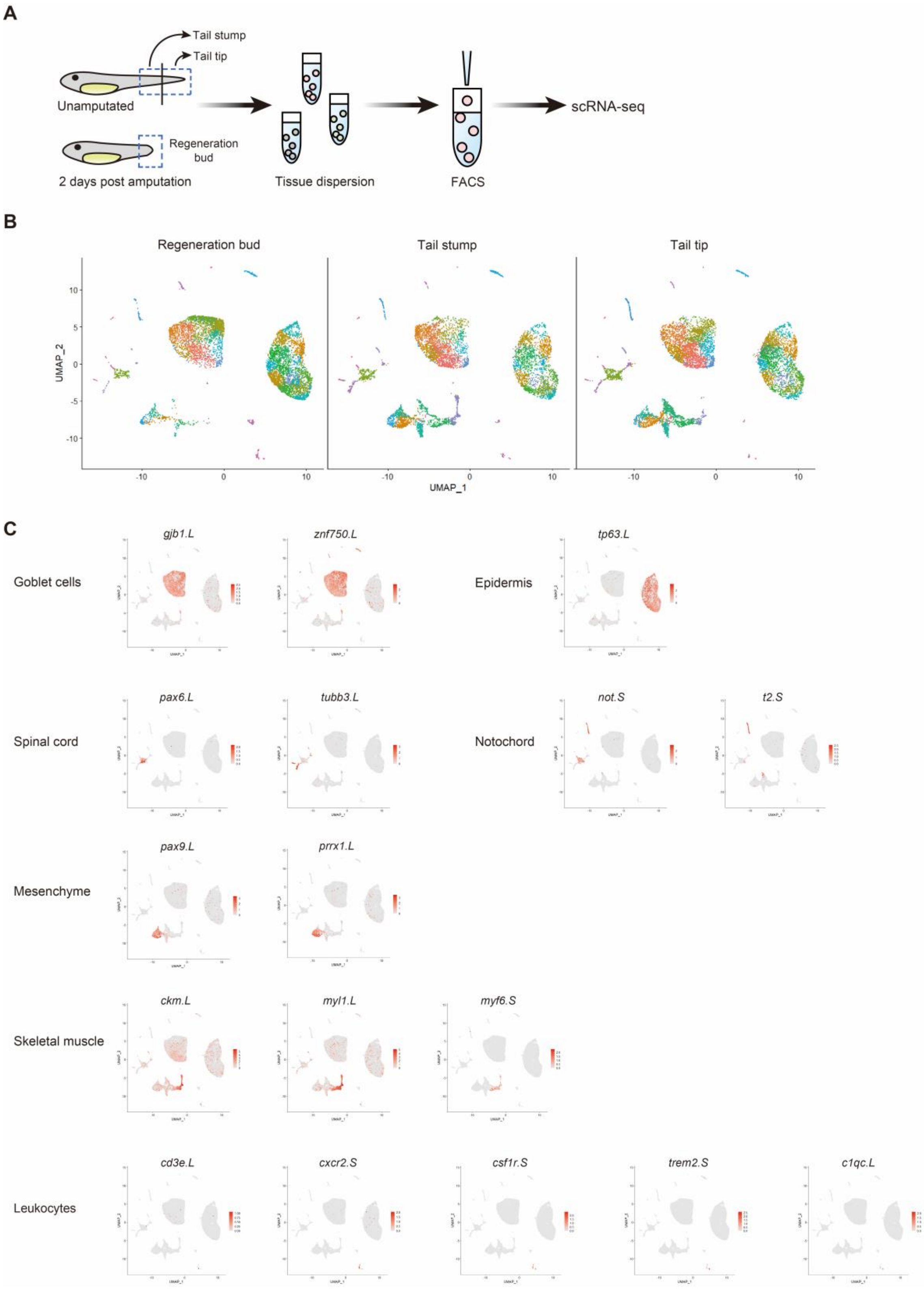

Development • Supplementary information

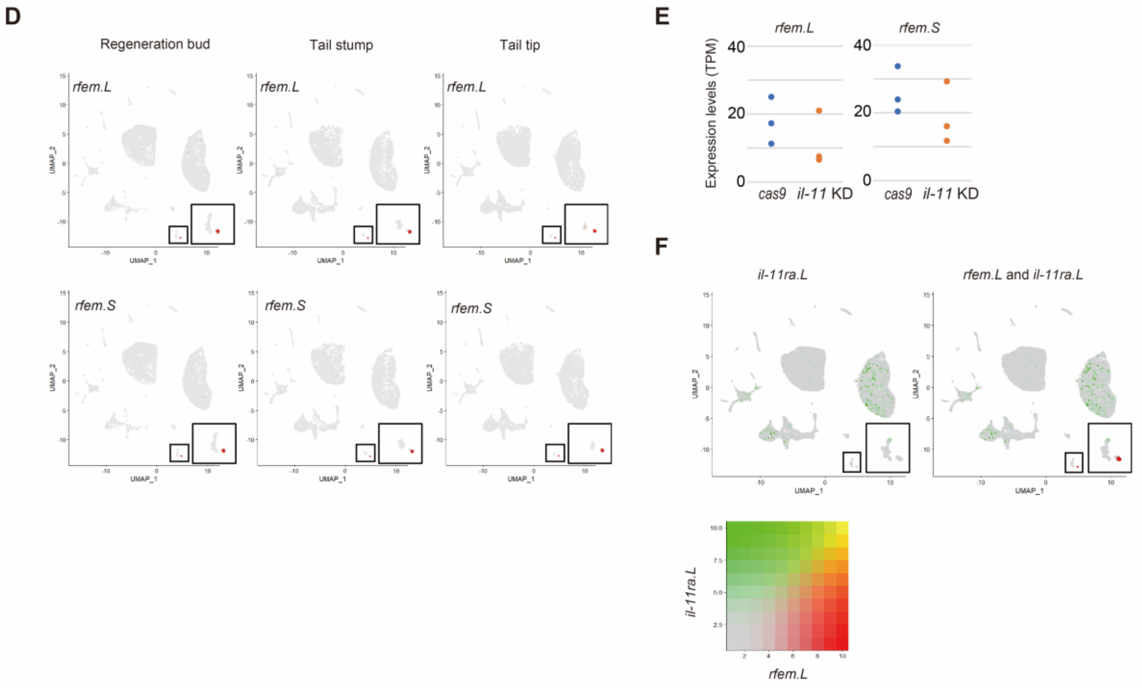

Fig. S3. *rfem* is expressed in a subset of leukocytes.

(A) Schematic drawing of the sample preparation for scRNA-seq. (B) Cells of the regeneration bud, tail stump, and tail tip are plotted separately by UMAP. (C) Expression of genes used for annotation of cell types in clusters plotted by UMAP in which three samples were combined. Markers for goblet cells, epidermis, spinal cord, notochord, mesenchyme, and skeletal muscle are based on a previous study [S2]. Markers for leukocytes are based on; *cd3e.L* [S2], *cxcr2.S* [S2], *csf1r.S* [S3], [S4] [S5], *trem2.S*[S6], [S7], and *c1qc.L* [S8], [S9], [S10]. (D) (upper) *rfem.L* and (lower) *rfem.S* expression in each sample. Expressing cells are indicated by red dots. (E) Expression levels of *rfem.L* and *rfem.S* in tail stumps at 48 h after amputation of *il11* KD and control tadpoles in the RNA-seq of a previous study (Tsujioka *et al.*, 2017) are shown in TPM. *n*=3. (F) Expression of (left) *il-11ra.L* and (right) *rfem.L* merged with the expression of *il-11ra.L* is shown in a UMAP plot in which three samples were combined. Expression of *il-11ra.L* and *rfem.L* is plotted in green and red, respectively. No cells coexpressing *il-11ra.L* and *rfem.L* were detected.

Development • Supplementary information

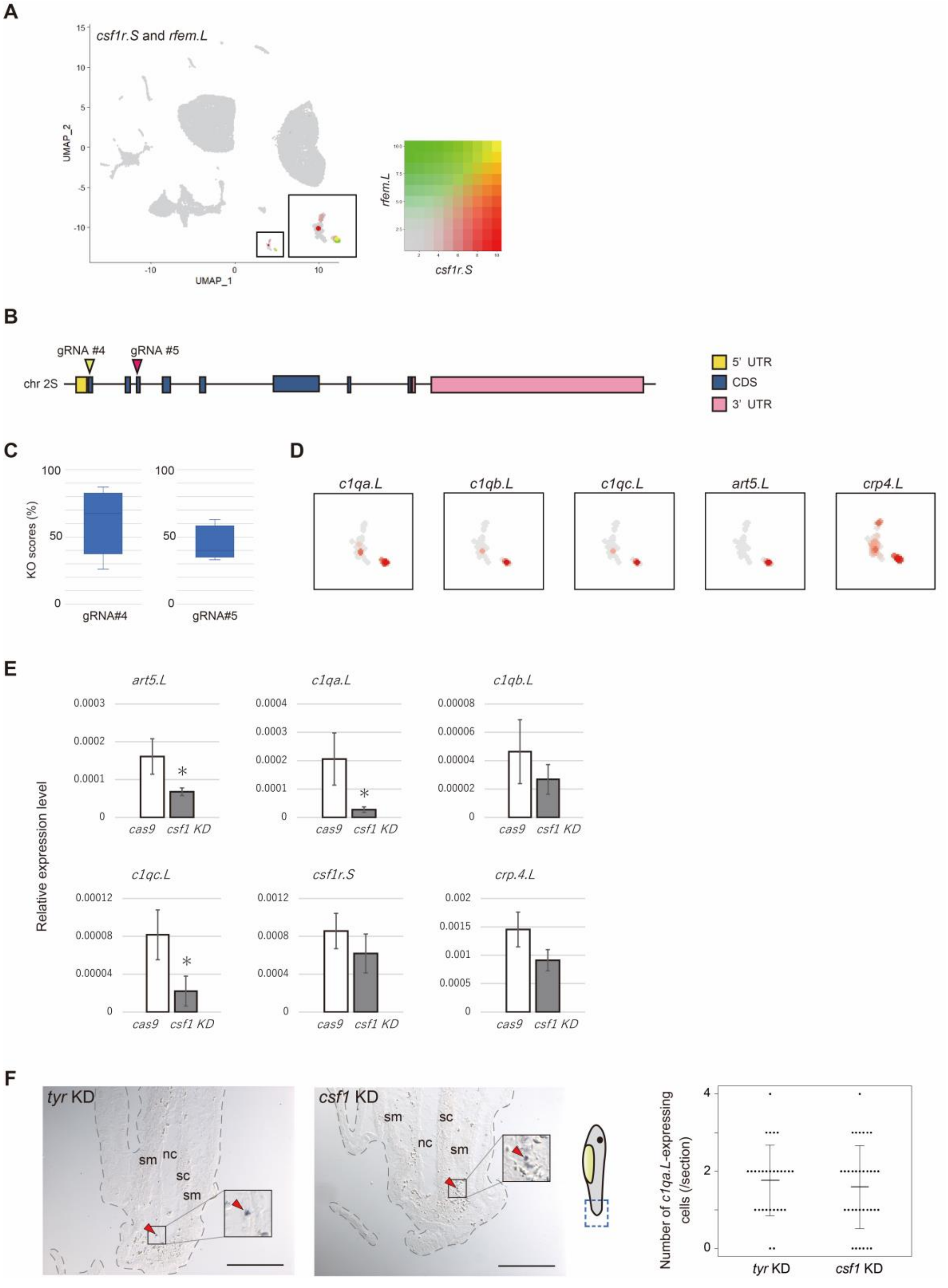

Development • Supplementary information

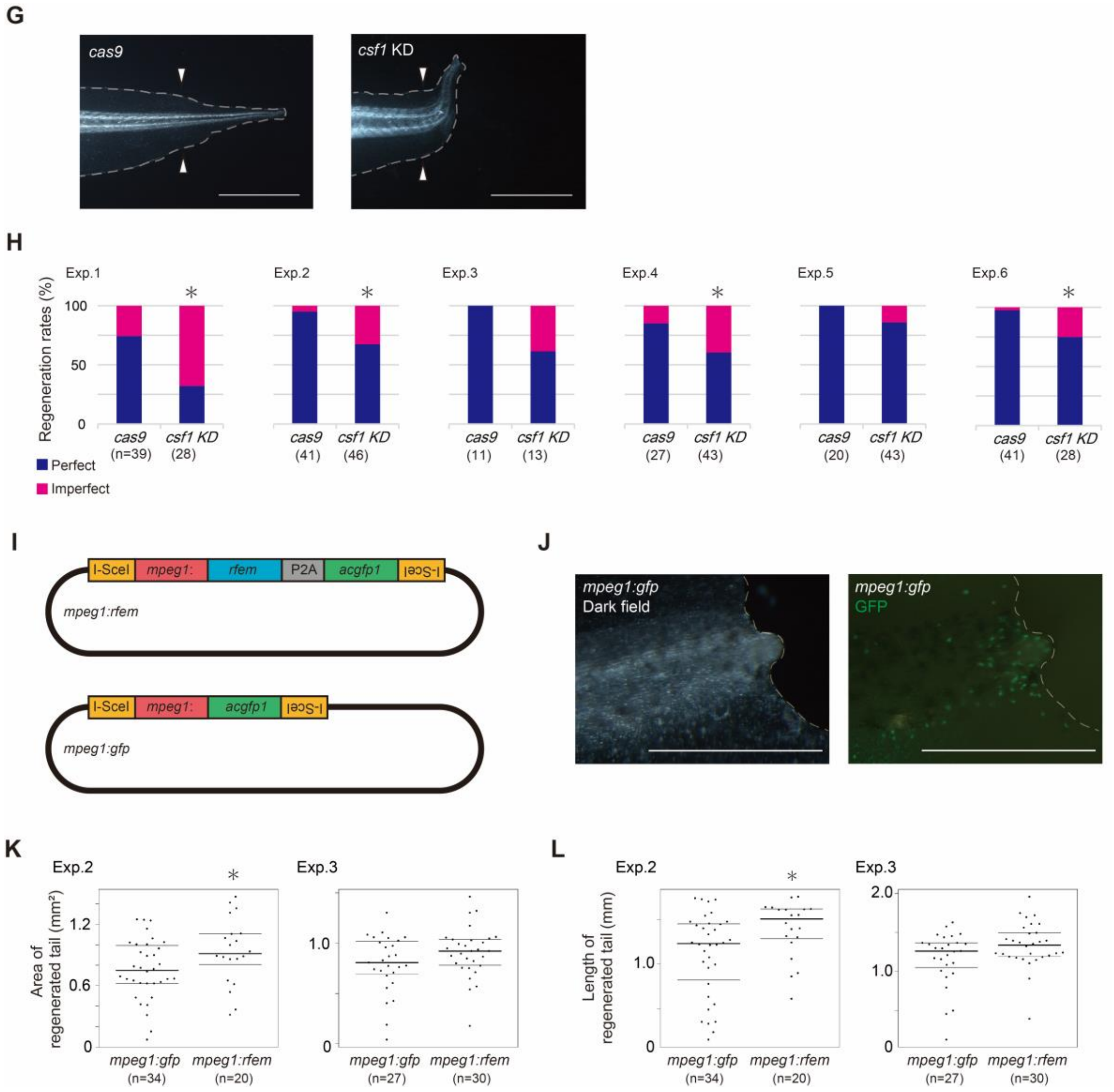

Fig. S4. KD of *csf1* impaired tail regeneration.

(A) Expression of *csf1r.S* and *rfem.L* is plotted in red and green, respectively. Co-expressing cells are plotted in yellow. (B) Target sites of guide RNAs used for KD of *csf1* are indicated by the arrowheads. (C) Box plots of KO scores of the gRNA #4 and #5 target site in *csf1.S* estimated using genomic DNA extracted from the amputated tail of each *csf1* KD tadpole. *n*=8. (D) Expression of genes indicated on the plots in the leukocyte cluster are plotted in red. (E) Relative expression levels of genes indicated on the graphs in the tail stumps at 24 h after amputation in tadpoles from *cas9* and *csf1* KD groups. Vertical axes represent relative expression levels normalized by those of *ef1α*. Mean ± s.d, $n = 3$. *P<0.05, Welch's *t* test. (F) (Left) Representative images of *in situ* hybridization of *c1qa.L* on sagittal sections of tails from 1 dpa *tyr* KD and *csf1* KD tadpoles. Red arrowheads indicate signals. Insets in the figures are magnified images of the boxed areas. Boxes with blue dashed lines in the schematic

Development • Supplementary information

diagrams indicate the location of the sections. nc, notochord; sc, spinal cord; sm, skeletal muscle. Scale bars: 200 μm. (Right) Number of *c1qa.L*-expressing cells in sections of 1 dpa tails of *tyr* KD (29 sections from 14 individuals) or *csf1* KD (34 sections from 12 individuals) tadpoles. Mean ± s.d., not significant in Welch's *t* test. (G) Examples of abnormal tail regeneration observed in the *csf1* KD group. Gray dashed line shows the outline of the tail. Arrowheads indicate amputation site. Scale bars: 2 mm. (H) Regeneration rates of *cas9* and *csf1* KD groups. Tadpoles from each experimental group were classified into 2 groups depending on the morphology of the regenerated tails: perfect or imperfect (see Methods). Six experiments (Exp. 1 to 6) were performed independently. The numbers of individuals in each experimental group are indicated below each graph. *P< 0.05 versus *cas9,* Fisher's exact test. (I) Schematic drawing of the rescue construct. (Upper) *rfem.L* coding sequence joined to P2A and *acgfp1* coding sequence was placed downstream of the zebrafish *mpeg1* promoter, which was flanked by 2 I-*Sce*I sites (*mpeg1:rfem*). To prevent gene editing of the rescue construct, synonymous substitutions were introduced into the *rfem.L* sequence at the gRNA#1 and #2 target sites. (Lower) A control construct having only the *acgfp1* coding sequence downstream of the *mpeg1* promoter (*mpeg1:gfp*). (J) Representative images of GFP-expressing cells in regenerated tails at 1 dpa in *rfem* KD tadpoles co-injected with the *mpeg1:gfp* (control). Gray dashed lines indicate the outline of the tail. Scale bars: 500 μm. (K and L) Measured area and length of regenerated tails at 7 dpa in control and *rfem*-rescued groups. The numbers of individuals in each experimental group are indicated below each graph. The horizontal lines indicate the 25th, 50th, and 75th percentiles, respectively. *P < 0.05, Welch's t test.

Development • Supplementary information

Table S1. Genes identified by the screening.

ADP-ribosyltransferase 5.L (art5.L)
complement C1q C chain.L (c1qc.L)
Xetrov90002578m.L (rfem.L)
Xetrov90002579m.S (rfem.S)

Table S2. (Upper) Numbers of injected embryos and normally developed tadpoles, and ratios of normally developed tadpoles. (Lower) Numbers of tadpoles in the *tyr* KD and *rfem* KD groups classified into each category.

Normal development ratio								
Exp.		Injected embryos	Normally developed tadpoles	Normal development ratio (%)		Injected embryos	Normally developed tadpoles	Normal development ratio (%)
1	*tyr* KD	125	39	31	*rfem* KD	120	35	29
2		98	64	65		129	91	71
3		64	41	64		53	37	70

Numbers of tadpoles in *tyr* KD and *rfem* KD groups classified into each category.								
Exp.		Perfect	Imperfect	Total		Perfect	Imperfect	Total
1*	*tyr* KD	16	12	18	*rfem* KD	9	9	18
2*		15	4	19		12	15	27
3		18	4	22		14	7	21

*P< 0.05, Fisher's exact test.

Development • Supplementary information

Table S3. Genes with significantly altered expression levels at the amputation site by *rfem* KD.

GeneID	Putative mouse homolog	log_2(FC)	padj
loc100487364.L	No significant similarity found	-1.18	2.94E-06
Xelaev18047455m.g	*hemoglobin Y, beta-like embryonic chain*	-1.07	2.94E-06
Xelaev18003021m.g	No significant similarity found	-4.84	1.61E-05
Xelaev18045084m.g	*hemoglobin Y, beta-like embryonic chain*	-1.08	1.61E-05
Xelaev18047464m.g	*hemoglobin X, alpha-like embryonic chain in Hba complex*	-0.84	0.00113
Xelaev18012120m.g	*uromodulin*	-1.15	0.00776

log_2(FC), log_2 transformed fold-change of expression level in the *rfem* KD group compared with the control group; padj, adjusted p value.

Table S4. (Upper) Numbers of injected embryos and normally developed tadpoles, and ratios of normally developed tadpoles. (Lower) Numbers of tadpoles in the *cas9* and *csf1* KD groups classified into each category.

Normal development ratio								
Exp.		Injected embryos	Normally developed tadpoles	Normal development ratio (%)		Injected embryos	Normally developed tadpoles	Normal development ratio (%)
1	*cas9*	78	40	51	*csf1*	160	66	41
2		74	47	64	*KD*	118	80	68
3		59	11	19		62	15	24
4		157	80	51		189	100	53
5*		156	72	46		173	111	64
6		145	77	53		173	92	53

Numbers of tadpoles in *cas9* and *csf1* KD groups classified into each category.								
Exp.		Perfect	Imperfect	Total		Perfect	Imperfect	Total
1*	*cas9*	29	10	39	*csf1*	9	19	28
2*		39	2	41	*KD*	31	15	46
3		11	0	11		8	5	13
4*		23	4	27		26	17	43
5		20	0	20		37	6	43
6*		40	1	41		21	7	28

*$P < 0.05$, Fisher's exact test.

Table S5. Normal development ratios of *rfem* KD tadpoles co-injected with *mpeg1:gfp* (control) or *mpeg1:rfem* constructs.

Exp.		Injected embryos	Normally developed tadpoles	Normal development ratio (%)		Injected embryos	Normally developed tadpoles	Normal development ratio (%)
1	*mpeg1:gfp*	89	24	27	*mpeg1:rfem*	77	29	38
2*		102	51	50		112	73	65
3*		86	28	33		81	40	50

*$P < 0.05$, Fisher's exact test.

Table S6. Primer and gRNA target sequences used in each experiment.

**qRT-PCR primer**	
ef1a qRT-PCR F	GGAACGGTGACAACATGC
ef1a qRT-PCR R	AGGCAGACGGAGAGGCTTA
rfem.L/S qRT-PCR F	AAGTTGACAAAGGAGCTTGGG
rfem.L/S qRT-PCR R	ACTACAGTTTGATAGGTTTGATAGGC
csf1.S qRT-PCR F	AGATGCCACTCTACCCTGCT
csf1.S qRT-PCR R	GGCTGCACCCCATGAATAGT
art5.L qRT-PCR F	CTCCAGCCCATAACTGCACA
art5.L qRT-PCR R	GAGAAGGTCCACTTGATCCCG
c1qa.L qRT-PCR F	GAACTGCCCTTCTGCTCAC
c1qa.L qRT-PCR R	TCTGGTGCCAAACACACG
c1qb.L qRT-PCR F	GGGCACCTCTGTGTGAATA
c1qb.L qRT-PCR R	CCAAACGGACTCGTCTTTC
c1qc.L qRT-PCR F	AGGGACCGACAGGACTTG
c1qc.L qRT-PCR R	AGGGACCGACAGGACTTG
crp.4.L qRT-PCR F	AAGGTTGATCCCACTGCTG
crp.4.L qRT-PCR R	CCATCGATCCAGAGCTCA
***in situ* hybridization**	
rfem.L in situ F	GCCAGTCACACCATTCTGACAT
rfem.L in situ R	TGAGGGTCTTGCTTCTTCCTTG
rfem.S in situ F	TCAGAGGACTGCAGGTCTAATCT
rfem.S in situ R	TCTGTGCGGGAGAATGATGAC
c1qa.L in situ F	GGCACCGCCTCCAAATCC
c1qa.L in situ R	TCTGTAGCAGTCATTGTGGGC
**gRNA target site + (PAM)**	
gRNA#1 (*rfem.L/S*)	GGGATCTACAGCTCAATTCC(AGG)
gRNA#2 (*rfem.L*)	GGAGCTTGGGTCCTGTATCA(GGG)
gRNA#3 (*rfem.S*)	GGCACAGCATCACCAGCGA(TGG)
gRNA#4 (*csf1.S*)	GAGTTAAAAAGGTCCGACG(GGG)
gRNA#5 (*csf1.S*)	GCAGATGCTTGCTTCGTCCG(AGG)
gRNA#6 (*tyr.L*/S)	GGCTCCATGTCTTCCGTCCA(AGG)
gRNA#7 (*tyr.L*/S)	GGCCCACTGCTCAGAAACCC(TGG)
**ICE-Analysis**	
ICE-PCR-*rfem.L*-F	GCTCATTGTGAAGTGCCAGTC
ICE-PCR-*rfem.L*-R	TGATGAGGGTCTTGCTTCTTCC

Development • Supplementary information

Table S6 (continued). Primer and gRNA target sequences used in each experiment.

ICE-PCR-*rfem.S*-F	AGAGGACTGCAGGTCTAATCTC
ICE-PCR-*rfem.S*-R	GTTCTGTGCGGGAGAATGATG
ICE-PCR-*csf1.S*#4-F	GAGAAAAGCAACAACAGAATACG
ICE-PCR-*csf1.S*#4-R	CGACTCGTGCTTGTTACTGC
ICE-PCR-*csf1.S*#5-F	GCCTTTGTTCGTGGACATG
ICE-PCR-*csf1.S*#5-R	TGGCAAATGCTTGTGCTG
ICE-seq-gRNA#1	GCTCATTGTGAAGTGCCAGTC
ICE-seq-gRNA#2-L	TGATGAGGGTCTTGCTTCTTCC
ICE-seq-gRNA#2-S	AGAGGACTGCAGGTCTAATCTC
ICE-seq-gRNA#3	GTTCTGTGCGGGAGAATGATG
ICE-seq-gRNA#4	GAGAAAAGCAACAACAGAATACG
ICE-seq-gRNA#5	TGGCAAATGCTTGTGCTG

Development • Supplementary information

Supplemental references

[S1]. **Session, A.M., Uno, Y., Kwon, T., Chapman, J.A., Toyoda, A., Takahashi, S., Fukui, A., Hikosaka, A., Suzuki, A., Kondo, M., et al.** (2016). Genome evolution in the allotetraploid frog *Xenopus laevis. Nature* **538**, 336–343. 10.1038/nature19840.

[S2].**Aztekin, C., Hiscock, T.W., Marioni, J.C., Gurdon, J.B., Simons, B.D., and Jullien, J.** (2019). Identification of a regeneration-organizing cell in the *Xenopus* tail. *Science* **364**, 653–658. 10.1126/science.aav9996.

[S3].**Stanley, E.R., and Chitu, V.** (2014). CSF-1 Receptor Signaling in Myeloid Cells. *Cold Spring Harb. Perspect. Biol.* **6**, a021857. 10.1101/cshperspect.a021857.

[S4].**MacDonald, K.P.A., Rowe, V., Bofinger, H.M., Thomas, R., Sasmono, T., Hume, D.A., and Hill, G.R.** (2005). The Colony-Stimulating Factor 1 Receptor Is Expressed on Dendritic Cells during Differentiation and Regulates Their Expansion. *J. Immunol.* **175**, 1399–1405. 10.4049/jimmunol.175.3.1399.

[S5].**Kuil, L.E., Oosterhof, N., Ferrero, G., Mikulášová, T., Hason, M., Dekker, J., Rovira, M., van der Linde, H.C., van Strien, P.M.H., de Pater, E., et al.** (2020). Zebrafish macrophage developmental arrest underlies depletion of microglia and reveals Csf1r-independent metaphocytes. *eLife.* **9**, e53403. 10.7554/eLife.53403.

[S6].**Hickman, S.E., and El Khoury, J.** (2014). TREM2 and the neuroimmunology of Alzheimer's disease. *Biochem. Pharmacol.* **88**, 495–498. 10.1016/j.bcp.2013.11.021.

[S7].**Ford, J.W., and McVicar, D.W.** (2009). TREM and TREM-like receptors in inflammation and disease. *Curr. Opin. Immunol.* **21**, 38–46. 10.1016/j.coi.2009.01.009.

[S8].**van de Bovenkamp, F.S., Dijkstra, D.J., van Kooten, C., Gelderman, K.A., and Trouw, L.A.** (2021). Circulating C1q levels in health and disease, more than just a biomarker. *Mol. Immunol.* **140**, 206–216. 10.1016/j.molimm.2021.10.010.

[S9]**.Chen, G., Tan, C.S., Teh, B.K., and Lu, J.** (2011). Molecular Mechanisms for Synchronized Transcription of Three Complement C1q Subunit Genes in Dendritic Cells and Macrophages. *J. Biol. Chem.* **286**, 34941–34950. 10.1074/jbc.M111.286427.

[S10].**Thi, M., Tran, N., Hamada, M., Jeon, H., Shiraishi, R., Asano, K., Hattori, M., Nakamura, M., Imamura, Y., Tsunakawa, Y., et al.** (2017). MafB is a critical regulator of complement component C1q. *Nat. Commun.* **8**, 1700. 10.1038/s41467-017-01711-0.
