# Supplementary material for: regeneration factors expressed on myeloid expression in macrophage-like cells is required for tail regeneration in Xenopus laevis tadpoles
Source: Development. 2023 Jul 31;150(15):dev200467. doi: 10.1242/dev.200467 (PMC10445729; doi:10.1242/dev.200467)
Supplement: Supplementary file 1 [file develop-150-200467_review_history.pdf]

regeneration factors expressed on myeloid expression in macrophage-like cells is required for tail regeneration in *Xenopus laevis* tadpoles

Momoko Deguchi, Taro Fukazawa and Takeo Kubo
DOI: 10.1242/dev.200467

Editor: Ken Poss

Review timeline
Original submission: 14 December 2021
Editorial decision: 26 January 2022
First revision received: 20 December 2022
Editorial decision: 1 February 2023
Second revision received: 10 June 2023
Accepted: 4 July 2023

Original submission

First decision letter

MS ID#: DEVELOP/2021/200467

MS TITLE: *csf1* dependent leukocytes that express *rplf* are required for tail regeneration in *Xenopus laevis* tadpole

AUTHORS: Momoko Deguchi, Taro Fukazawa, and Takeo Kubo

I have now received all the referees' reports on the above manuscript, and have reached a decision. The referees' comments are appended below, or you can access them online: please go to BenchPress and click on the 'Manuscripts with Decisions' queue in the Author Area.

As you will see, the referees express considerable interest in your work, but have some significant criticisms and recommend a substantial revision of your manuscript before we can consider publication. If you are able to revise the manuscript along the lines suggested, which will probably involve further experiments, I will be happy receive a revised version of the manuscript. Your revised paper will be re-reviewed by one or more of the original referees, and acceptance of your manuscript will depend on your addressing satisfactorily the reviewers' major concerns. Please also note that Development will normally permit only one round of major revision.

We are aware that you may be experiencing disruption to the normal running of your lab that make experimental revisions challenging. If it would be helpful, we encourage you to contact us to discuss your revision in greater detail. Please send us a point-by-point response indicating where you are able to address concerns raised (either experimentally or by changes to the text) and where you will not be able to do so within the normal timeframe of a revision. We will then provide further guidance. Please also note that we are happy to extend revision timeframes as necessary.

Please attend to all of the reviewers' comments and ensure that you clearly highlight all changes made in the revised manuscript. Please avoid using 'Tracked changes' in Word files as these are lost in PDF conversion. I should be grateful if you would also provide a point-by-point response detailing how you have dealt with the points raised by the reviewers in the 'Response to Reviewers' box. If you do not agree with any of their criticisms or suggestions please explain clearly why this is so.

© 2023. Published by The Company of Biologists under the terms of the Creative Commons Attribution License (https://creativecommons.org/licenses/by/4.0/).

Reviewer 1

Advance summary and potential significance to field

Degushi et al present an interesting manuscript addressing the mechanisms behind tail regeneration in Xenopus laevis, describing the involvement of previously uncharacterized genes (rplf.L/S) in this process. Further, they suggest that expression of said genes by csf1-dependent leukocyte population is important for regeneration.

These findings are novel and of great interest to the community. However, the main claims are not fully supported by the available data, and further experimental evidence is required prior to further consideration.

Comments for the author

Major points:
- Lack of in-depth characterization of KD models. In order to strengthen the authors' arguments, I would suggest the following further analyses:
a) The use of mosaic F0 crispants (rplf or csf1) without thorough analysis of the mutations induced makes data interpretation difficult, and the regeneration data shown reveal quite considerable differences between experiments. The HMA assay used would only show that some mutations have occurred at the site of interest but will not show the nature of these (e.g. many could be non-frameshift mutations). This could be approached through deep sequencing analysis from the different experimental batches, and phenotype-genotype matching. This is critical as the authors work with F0 animals.
b) While the authors present cas9 injection as negative control, a more appropriate control would be cas9 co-injection with a gRNA that does not have an impact in regeneration/development (e.g. tyrosinase or similar). At present, it cannot be ruled out that the regeneration defects arise due to impurities derived from the RNA purification protocol or similar.
c) The mutagenicity of one gRNA used (gRNA4 for csf1.S) was not assessed.
d) The authors should also provide clarity on how they select crispants for experimental analysis – are any gRNA-injected, surviving, healthy tadpoles used, or are all tadpoles first validated at least by HMA?
e) The authors call their F0 crispants rpl knockdowns, but do not actually validate that rpl factor expression is decreased in these crispants.
f) Therefore, it is clear that much more thorough analysis of the F0s is needed, in terms of both the mutations induced and the resulting phenotypes (e.g. qPCR/ISH/stainings for macrophages or factors).
- The analysis of regenerative efficacy by the binary 'perfect' or 'imperfect' metric is very limited, and timing of the effects in regeneration was not analysed. Further characterization of regeneration defects including time curves and in-depth analysis of regenerating tissues is required (eg. quantitative data for the size of the regenerated area, bone/cartilage stainings, ECM stainings, etc). Also, are the defects in regeneration corrected later on, or after a new amputation?
- Lack of support for the central claim that 'rplf expressing csf1-dependent leukocytes are required for tail regeneration: the work shows that regeneration required rplf, and independently that replies on csf1, but no strong evidence is presented to support the main claim. For this, cell type-specific rescue experiments should be conducted (e.g. expression of rplf.L/S under a leukocyte promoter as these approaches are available in xenopus – for example mpeg or ideally a promoter expressed in the csf1 leukocytes)
- Monocyte/macrophage quantification from csf1-KD tadpoles is required in order to address whether differences in gene expression (including rplf) and regeneration correlate with a decrease in the target leukocyte population.
- Overall, the images presented are of low resolution and TOO SMALL so that assessment is extremely difficult – this should be corrected Additional points:
- While it is understandably difficult to identify the function of uncharacterized genes with few homologues, the authors have presented no data on the function of the rpl genes. It would improve the study if the authors performed some simple additional experiments in the rpl KD tadpoles. For example, qPCR measurements of macrophage-specific genes could reveal if rpl

© 2023. Published by The Company of Biologists under the terms of the Creative Commons Attribution License (https://creativecommons.org/licenses/by/4.0/).

factors are essential for macrophage development, or alternatively for macrophage recruitment to regenerating tails.

- The naming of the rpl factors could be improved as rpl genes are ribosomal factors.
- considering the data currently presented, I would suggest changing the legend of Fig.4, as it does not show that the rplf-expressing cells are depleted in the csf1 KD tadpoles. The figure shows changes in gene expression detected in the whole tail-stump sample, and in order to claim that this lower expression is due to cell depletion, I would suggest providing rplf+ cell numbers in the csf1-KD tadpoles compared to their Cas9 controls.
- A few typos need correction, namely:

(1) Xetrov90002579m.S is annotated as Xetrov90002579m.L on Fig.S1B (at chr 1S)
(2) On line 20 a word might be missing between “type” and “supports”
(3) On line 22, it should be “afterwards” instead of “afterword”
(4) On line 99, I think the referenced figure should be Fig.S2A instead of Fig.S1A
(5) On line 315, the reference used is number 49, but there are 45 references, so it might be 39 instead.

Reviewer 2

Advance summary and potential significance to field

In this manuscript Deguchi et al investigate xenopus tail regeneration focusing on characterizing the mechanism by which IL11 regulates this mechanism. They first mine expression dataset to identify candidate gene with an IL11 dependent role in regeneration. They focus on rplf and show that it is expressed after amputation and that it shows a punctuated pattern in tadpole tail. They then perform crispr experiment to show that rplf is required for regeneration. By scRNA-seq rplf is seen expressed in leucocytes. Lastly they show that crispr of CSF1 impairs rgeneration as well as amputation mediated expression of rplf.

The author indicates that the previously described involvement of IL11 in regeneration is not cell autonomous and that this work aims at deciphering the IL11 target cells. Their previous work indicated that IL11 mRNA is found in progenitor cells. Here they propose that leucocytes could be IL11 responsive cells. However the expreriements do not allow to reach such conclusions.

Comments for the author

1/ rplf as an amputation induced gene.
The expression pattern by in situ is compatible with expression in leucocyte. However:
(1) Additional marker should be used to demonstrate that the cells are indeed leucocytes (i.e Paredes et al dev biol, 2015) or qtify rplf in situ in WT versus crispr CSF1 tadpole.
(2) The statement that an increase in rplf expressing cells upon amputation is observed need to be backed up by quantitation of in situ data. This is important in light of the fact that in scRNA-seq dataset while rplf is restricted to leucocyte cluster it does not seem to be expressed in a regeneration dependent manner (suppl fig 3). Maybe that the authors can also evaluate if rpfl expression level change in the in the leucocyte cluster in tail versus blastema?
2/ To address their initial question they need to perform crspr on IL11 and check if rplf is indeed not induced in leucocytes
3/As presented the crispr CSF1 KO is not very well linked to the rest of story. I would see it better as a tool to demonstrate that RFPL is expressed in leucocyte (see point 1). Indeed the role of leucocyte in regeneration is already documented (Aztekin et al, development 2020; Fukawaza et al development ,2009) so novelty here is not so strong. At least it should be put in perspective with these other studies.
4/In general the crispr phenotypte should be better analysed/presented.
-As mentioned by authors the crispr F0 assay shows a mosaic effect. The authors present for each crispr target 8 different experiments with only a subset showing effect (i.e 5/8 for the rplf crispr assay). I assume the reason for heterogeneity is difference in crispr efficiency, but there is no data to back this up. The regeneration assay is well suited to measure crispr efficiency from individual tadpoles (using amputated tail material), for example using a T7-Endonuclease I assay to quantify

© 2023. Published by The Company of Biologists under the terms of the Creative Commons Attribution License (https://creativecommons.org/licenses/by/4.0/).

the percentage of heteroduplex, or using TIDE . This would be a more convincing way to link crispr efficiency to regeneration outcome.
-Similarly it would be important to check that those tadpoles excluded from the analysis (abnormal) are not those showing the strongest crispr KD of rplf (indeed rplf KD show more abnormality than control)
-It is not clear why the assessment of regeneration is shown with percentage of imperfect/perfect. Why are the authors not using the more detailed RI index T7-Endonucléase Iused in previous work from this group (i.e Fukawaza et al development ,2009)

First revision

Author response to reviewers' comments

Reviewer 1

We are grateful to Reviewer 1 for the useful suggestions that have helped us to considerably improve our paper. As indicated in the responses that follow, we have taken all these comments and suggestions into account in the revised version of our manuscript.

1. -Lack of in-depth characterization of KD models. In order to strengthen the authors' arguments, I would suggest the following further analyses:

1.a) The use of mosaic F0 crispants (rplf or csf1) without thorough analysis of the mutations induced makes data interpretation difficult, and the regeneration data shown reveal quite considerable differences between experiments. The HMA assay used would only show that some mutations have occurred at the site of interest, but will not show the nature of these (e.g. many could be non-frameshift mutations). This could be approached through deep sequencing analysis from the different experimental batches, and phenotype-genotype matching. This is critical as the authors work with F0 animals.

Thank you for this comment. We agree that quantitative assessment of gene-editing efficiency is preferable over HMA. Accordingly, we performed genotyping of F0 crispants using Inference of CRISPR Edits-analysis (ICE-analysis), a method of estimating the proportion of gene-edited clones in genomic DNA (Hsiau et al., bioRxiv 251082, 2019). We replaced the previous figure showing the HMA results (Fig. S2C and S4C in the previous version) with new figures showing the results of ICE analysis as Fig. S2C and Fig. S4C, and described the results in lines 110-114 on p.5 and lines 174-175 on p.8 in the revised version for the *rfem* knock down (KD) and *csf1* KD experiments, respectively.
Regarding *rfem* (the gene name *rplf* was changed to *rfem* in the revised manuscript, according to Reviewer 1's comment-7), *rfem.L/S* are expressed in a subset of leukocytes; thus, it would be preferable to perform the phenotype-genotype matching using genomic DNA of the leukocyte fraction isolated from each individual. Unfortunately, however, we did not think we could perform the experiment with the current methodologies within a reasonable timeframe. Therefore, we tried to detect a correlation between the phenotype (area of the regenerated tail) and genotype using ICE analysis with genomic DNA of the peripheral blood cell (PBC) fraction isolated from each individual, but no correlation was detected, perhaps because of the small proportion of the leukocyte subset in the PBC fraction. We described this result in lines 160-164 on p.7, and added a new Fig. S2I. We did not think that the batch effect (differences between experiments) could be addressed with phenotype-genotype matching using the PBC fraction, however, and therefore did not repeat the experiment, and instead dealt with this issue in another way. Reviewer 1's concern is that our previous data of regeneration outcome assessments showed considerable differences between experiments. In the revised version, we measured both the area and length of the regenerated tails to assess the regenerative capacity, according to the comments of Reviewers 1 and 2. We detected a significant reduction in the regenerative capacity in *rfem* KD group with reproducibility; all 3 experiments showed a significant reduction in the area and length of the regenerated tails. Therefore, this new measurement minimized the batch effects between experiments on the regenerative capacity assessment. We described this result in lines 124-125 on

© 2023. Published by The Company of Biologists under the terms of the Creative Commons Attribution License (https://creativecommons.org/licenses/by/4.0/).

p.6, and added new Figs. 2D, 2E, and S2F-H.

Regarding *csf1*, the phenotype-genotype matching in the *csf1* KD experiment would be better if performed with genomic DNA from a cell fraction that expresses *csf1*, but we have no methodologies to isolate the fraction. In the previous manuscript, we utilized *csf1* to assess the relationship between *rfem* and macrophage-lineage cells by *csf1* KD and subsequent depletion of the *csf1*-dependent leukocytes; in the revised version, we performed forced expression of *rfem.L* in macrophage-like cells of *rfem* KD individuals under control of the zebrafish *mpeg1* promoter, as suggested by Reviewer 1, and found that the forced expression recovered regeneration outcomes of the *rfem* KD individuals. This result strongly indicates that defects in tail regeneration in the *rfem* KD group were largely due to the loss of *rfem* function in macrophage-like cells. We described this result in lines 39-41 on p.2 (Abstract) and lines 187-197 on p.8-9 (Results and Discussion), and added new Figs. 4C-F, and S4H-K in the revised version. We think that the result of this rescue experiment further strengthens our argument that *rfem* expression in macrophage-lineage cells is required for tail regeneration, even without having performed the phenotype-genotype matching in the *csf1* KD experiment, which would be technically difficult for us.

We hope that our responses satisfactorily address the reviewer's concern.

1.b) While the authors present cas9 injection as negative control, a more appropriate control would be cas9 co-injection with a gRNA that does not have an impact in regeneration/development (e.g. tyrosinase or similar). At present, it cannot be ruled out that the regeneration defects arise due to impurities derived from the RNA purification protocol or similar.

Thank you for this comment. Accordingly, we re-performed the *rfem* KD experiments with a group that was co-injected with *cas9* mRNA and gRNA targeting *tyrosinase* (*tyr* KD) as a negative control, and observed that *rfem* KD groups showed a significant reduction in their regenerative capacity compared with the *tyr* KD groups with reproducibility, indicating that the regeneration defects in the *rfem* KD group were not due to a problem with the gRNAs. We replaced Figs. 2B, C and S2B, D in the previous version showing tadpoles injected with only *cas9* mRNA as a negative control, with new Figs. 2B-E, and S2B, D-H in the revised version. Regarding *csf1* KD experiments, as mentioned in our response to Reviewer 1's comment-1a, we performed the *csf1* KD experiments in the previous version to assess the relationship between *rfem* and macrophage-lineage cells; however, the macrophage-like cell- specific rescue experiment provided more direct evidence. In addition, the *tyr* KD experiment mentioned above lowered the possibility of problems with our gRNAs. Considering this along with the limited timeframe for revision, we think that the rescue experiment strengthens our argument without the need to re-perform the *csf1* KD experiments using *tyr* KD as a negative control. Regarding the *csf1* KD experiments that we newly performed for the revision (*rfem in situ* hybridization on *csf1* KD tadpoles; mentioned in our response to Reviewer 1's comment-8), we used the *tyr* KD group as a negative control, according to Reviewer 1's comment. We described this result in lines 37-39 on p.2 (Abstract) and lines 180-183 on p.8 (Results and Discussion), and added a new Fig. 4B in the revised version.

1.c) The mutagenicity of one gRNA used (gRNA4 for csf1.S) was not assessed.

Thank you for this comment. Accordingly, we performed an assessment of the mutagenicity of gRNAs, including gRNA4 for *csf1.S,* using ICE analysis as described in our responses to Reviewer 1's comment-1a. We described this result in lines 174-175 on p.8, and added a new Fig. S4C in the revised version.

1.d) The authors should also provide clarity on how they select crispants for experimental analysis - are any gRNA-injected, surviving, healthy tadpoles used, or are all tadpoles first validated at least by HMA?

Thank you for this comment. For experimental analysis, we used all mRNA and gRNA- injected, surviving, and healthy tadpoles at 4 days post fertilization. Accordingly, we added this description to lines 317-318 on p.14 in the revised version (Materials and Methods section).

1.e) The authors call their F0 crispants rpl knockdowns, but do not actually validate that rpl

© 2023. Published by The Company of Biologists under the terms of the Creative Commons Attribution License (https://creativecommons.org/licenses/by/4.0/).

factor expression is decreased in these crispants.

Thank you for this comment. Regarding the expression analysis of *rfem*, there is no available antibody for *X. laevis* Rfem, making it difficult to assess the expression level of Rfem protein in the F0 crispants. A gene mutation induced by gene editing does not always reduce the mRNA expression level of the gene, and we did not think that quantification of the mRNA expression of *rfem* in the *rfem* F0 crispants would address this concern expressed by Reviewer 1. Alternatively, we performed ICE analysis and estimated the knockout (KO) score, i.e., the proportion of cells that have with either a frameshift-inducing insertion/deletion (indel) or an indel ≥ 21 bp at the target site of each gRNA (Hsiau et al., 2019). We detected such mutations in most of the randomly selected *rfem* F0 crispants, suggesting that most of F0 crispants were mosaics containing KO cells. As mentioned in our response to Reviewer 1's comment-1a, we replaced the previous figure showing the HMA results (Fig. S2C in the previous version) with a new figure showing the results of the ICE analysis of the *rfem* KD experiment as Fig S2C, and described the results in lines 110-114 on p.5 in the revised version.

1.f) Therefore, it is clear that much more thorough analysis of the F0s is needed, in terms of both the mutations induced and the resulting phenotypes (e.g. qPCR/ISH/stainings for macrophages or factors).

Thank you for this comment. Regarding analysis of the mutations induced, as described in our response to Reviewer 1's comment-1e, we performed a quantitative estimation of the proportion of KO cells in the F0 crispants using ICE analysis and the results indicated that most of the F0 crispants are mosaics containing KO cells. Regarding analysis of the phenotypes, as described in our response to Reviewer 1's comment-1a, we measured the area and length of the regenerated tails, and observed a significant reduction in the size and length of the regenerated tails in *rfem* KD tadpoles with reproducibility between experiments.

In addition, we newly performed RNA-sequencing (RNA-seq) with tail stumps of *rfem* KD tadpoles 48 hours post amputation. We detected 6 differentially expressing genes (DEGs) whose expression was significantly reduced in *rfem* KD samples, including 3 *hemoglobin* genes, suggesting a possibility that *rfem* KD affects wound repair, including blood vessel formation, although angiogenic factors were not detected as DEGs. We described this result briefly in lines 131-134 on p.6 and added a new Table S3 in the revised version. We found that the factors reported to be associated with macrophage function were not included in DEGs, suggesting that macrophage-accumulation at the wound site was not affected by *rfem* KD. The result also suggested a possibility that *rfem* KD does not affect transcription levels of these factors in macrophages, consistent with the fact that Rfem has no known DNA-binding domains and is not thought to be a transcription factor. The main scope of this article is that *rfem* expression in macrophage-like cells is required for tail regeneration, and the Rfem function in these cells is beyond the scope of the present study. We plan to proceed with this analysis in the future.

2. -The analysis of regenerative efficacy by the binary 'perfect' or 'imperfect' metric is very limited, and timing of the effects in regeneration was not analysed. Further characterization of regeneration defects including time curves and in-depth analysis of regenerating tissues is required (eg. quantitative data for the size of the regenerated area, bone/cartilage stainings, ECM stainings, etc). Also, are the defects in regeneration corrected later on, or after a new amputation?

Thank you for this comment. Accordingly, we measured the area and length of the regenerated tails to assess the regeneration outcomes. Area and length measurements were performed over time. In addition, the area of the muscle and notochord in the regenerated tails was measured. These analyses successfully detected regeneration defects in *rfem* KD groups with reproducibility. We described the results in lines 124-125 on p.6 and added new Figs. 2D-E, and S2F-H in the revised version.

We also performed follow-up observation of *rfem* KD tadpoles that showed poor regeneration outcomes for 1-2 months after amputation. We observed that the abnormal morphology of regenerated tails of *rfem* KD tadpoles was maintained as they grew, and these tadpoles showed poor regeneration outcomes after re-amputation. We described the results in lines 126-128 on p.6 and

© 2023. Published by The Company of Biologists under the terms of the Creative Commons Attribution License (https://creativecommons.org/licenses/by/4.0/).

added new Figs. S2J, K in the revised version.

3. -Lack of support for the central claim that 'rplf expressing csf1-dependent leukocytes are required for tail regeneration: the work shows that regeneration required rplf, and independently that replies on csf1, but no strong evidence is presented to support the main claim. For this, cell type-specific rescue experiments should be conducted (e.g. expression of rplf.L/S under a leukocyte promoter as these approaches are available in xenopus - for example mpeg or ideally a promoter expressed in the csf1 leukocytes)

We agree that this is an important point.

Accordingly, we performed a cell-type specific rescue experiment. We designed constructs that express *rfem.L* and *acgfp1* bicistronically, or only *acgfp1* under control of the zebrafish *mpeg1* promoter (*mpeg1:rfem* and *mpeg1:gfp* respectively) to perform macrophage-like cell-specific rescue in *rfem* KD tadpoles as suggested. The expression cassette was flanked by 2 I-*Sce*I meganuclease recognition sites to perform I-*Sce*I mediated transgenesis, which would facilitate the expression by stably inserting into the genome, in addition to the free constructs remaining in the cells of the tadpole at the time of tail amputation. We observed scattered GFP-expressing cells in regenerating tails of tadpoles injected with the construct, suggesting that the construct worked as expected. Compared with control individuals (*rfem* KD individuals co-injected with *mpeg1:gfp*), the area and length of the regenerated tails in the *mpeg1:rfem*-rescued *rfem* KD individuals 7 days post amputation (dpa) were significantly recovered, indicating that *rfem* expression in macrophage-like cells has an indispensable role in successful tail regeneration. We described the results in lines 39-41 on p.2 (Abstract) and lines 187-197 on p.8-9 (Results and Discussion) and added new Figs. 4C-F and S4H-K. We thank Reviewer 1 for the suggestion, which helped to strengthen our argument.
In the previous manuscript, we utilized *csf1* to assess the relationship between *rfem* and macrophage-lineage cells; we think this purpose of the *csf1* KD experiment was achieved with the rescue experiment. Thus, we moved some figures regarding *csf1* KD experiments to Supplementary information: Fig. 4B (quantification of macrophage factor gene expression in the *csf1* KD group), 4C (representative photos of regenerated tails of *csf1* KD tadpoles), and 4D (regeneration rates of *csf1* KD tadpoles) in the previous version, to Figs. S4E-G in the revised version, respectively. Fig. 4A in the previous version (schematic figure of *csf1* KD experiment design) was replaced with a new Fig. 4C (schematic figure of the cell type-specific rescue experiment design) in the revised version.

As we observed the result, we would like to change the title "*csf1* dependent leukocytes that express *rplf* are required for tail regeneration in *Xenopus laevis*" in the previous version, to a more suitable title that represents our argument supported by current results, as "*rfem* expression in macrophage-like cells is required for tail regeneration in *Xenopus laevis* tadpole" in the revised version, because there is no information and no experimental evidence of a relationship between *csf1*-dependent leukocytes and macrophage-like cells with zebrafish *mpeg1* promoter activity. We also revised the sentence "Our findings suggested that *csf1*-dependent leukocytes are necessary for tail regeneration, and *rplf.L/S* are required for the regeneration-promoting function of the leukocytes." in the Abstract of the previous manuscript to "Our findings suggest that *rfem.L/S* expression in macrophage-like cells is required for tail regeneration." in lines 41-42 on p.2 (Abstract) in the revised manuscript. The gene name "*rplf* (*regeneration-promoting-leukocytes factor*)" in the previous version was also changed to "*rfem* (*regeneration factors expressed on myeloid*)" in the revised version, according to Reviewer 1's comment-7.

4. -Monocyte/macrophage quantification from csf1-KD tadpoles is required in order to address whether differences in gene expression (including rplf) and regeneration correlate with a decrease in the target leukocyte population.

Thank you for this comment. We agree with that macrophage quantification on *csf1* KD tadpoles provides important information about the effect of *csf1* KD on macrophage- lineage depletion. We performed *in situ* hybridization of *rfem.L/S* on amputated tails of *csf1* KD and *tyr* KD (as a negative control, according to Reviewer 1's comment-1b), and detected a significant reduction in the numbers of *rfem*-expressing cells in the tail stumps of *csf1* KD tadpoles. We described the result in lines 37-39 on p.2 (Abstract) and lines 180-183 on p.8 (Results and Discussion) and added a new Fig. 4B in the revised version.

© 2023. Published by The Company of Biologists under the terms of the Creative Commons Attribution License (https://creativecommons.org/licenses/by/4.0/).

As mentioned in our response to Reviewer 1's comment-3, we observed that forced expression of *rfem* in macrophage-like cells of *rfem* KD tadpoles rescued their regeneration defects, providing direct evidence of *rfem* function in macrophage-like cells. We utilized *csf1* to assess the relationship between *rfem* and macrophage-lineage cells; we think the purpose of the *csf1* KD experiment was achieved with the rescue experiment. Considered together with the timeframe for revision, we think that our argument is strongly supported by the rescue experiment even without further analysis of *csf1* KD, which would require considerable time.

5. -Overall, the images presented are of low resolution and TOO SMALL so that assessment is extremely difficult - this should be corrected

Thank you for this comment. In the initial submission, the figures were low resolution to reduce the file size, which might have obscured the details. The figures in the revised version have a higher resolution, and show the details more clearly.

Additional points:

6. -While it is understandably difficult to identify the function of uncharacterized genes with few homologues, the authors have presented no data on the function of the rpl genes. It would improve the study if the authors performed some simple additional experiments in the rpl KD tadpoles. For example, qPCR measurements of macrophage-specific genes could reveal if rpl factors are essential for macrophage development, or alternatively for macrophage recruitment to regenerating tails.

Thank you for this comment. Accordingly, as mentioned in our response to Reviewer 1's comment-1f, we newly performed RNA-seq with tail stumps of *rfem* KD tadpoles. We detected 6 DEGs, including 3 *hemoglobin* genes, whose expression was significantly reduced in *rfem* KD samples, suggesting a possibility that *rfem* KD affects wound repair, including blood vessel formation. We described this result briefly in lines 131-134 on p.6 and added a new Table S3 in the revised version. The DEGs did not include factors reported to be associated with macrophage function, suggesting that macrophage accumulation at the wound site was not affected by *rfem* KD. It is possible that *rfem* KD does not affect transcription of these factors in macrophages, consistent with the fact that Rfem has no known DNA-binding domains and is thus not thought to be a transcription factor. Further analysis of *rfem* function requires considerable time, we did not think we could complete additional analysis within the limited timeframe for revision, but we plan to proceed with these analyses in the future.

7. -The naming of the rpl factors could be improved as rpl genes are ribosomal factors.

Thank you for your comment. Accordingly, we changed the gene name "*rplf* (*regeneration-promoting-leukocytes factor*)" in the previous version to "*rfem* (*regeneration factors expressed on myeloid*)" in the revised version.

8. -considering the data currently presented, I would suggest changing the legend of Fig.4, as it does not show that the rplf-expressing cells are depleted in the csf1 KD tadpoles. The figure shows changes in gene expression detected in the whole tail- stump sample, and in order to claim that this lower expression is due to cell depletion, I would suggest providing rplf+ cell numbers in the csf1-KD tadpoles compared to their Cas9 controls.

Thank you for this comment. Accordingly, we performed *in situ* hybridization of *rfem.L/S* on amputated tails of *csf1* KD and *tyr* KD (as negative control, according to Reviewer 1's comment-1b) tadpoles, and detected a significant reduction in the number of *rfem*-expressing cells at tail stumps of *csf1* KD tadpoles compared with that of *tyr* KD tadpoles. We described the result in lines 37-39 on p.2 (Abstract) and lines 180-183 on p.8 (Results and Discussion) and added new Fig. 4B in the revised version.

9. -A few typos need correction, namely:

(1) Xetrov90002579m.S is annotated as Xetrov90002579m.L on Fig.S1B (at chr 1S)

© 2023. Published by The Company of Biologists under the terms of the Creative Commons Attribution License (https://creativecommons.org/licenses/by/4.0/).

Thank you for this comment. We corrected the typo in Fig.S1B in the revised version.

(2) On line 20 a word might be missing between "type" and "supports"

Thank you for this comment. The sentence "we aimed to screen genes that function in a certain cell type supports tail regeneration under Il-11 signaling …" in the previous version was revised to "we screened for genes that support tail regeneration under Il-11 signaling in a certain cell type…" in lines 31-32 on p.2 in the revised version.

(3) On line 22, it should be "afterwards" instead of "afterword"

Thank you for this comment. We revised the sentence to "referred to hereafter as *regeneration factors expressed on myeloid.L* [*rfem.L*] and *rfem.S*" in line 33-34 on p.2 in the revised version.

(4) On line 99, I think the referenced figure should be Fig.S2A instead of Fig.S1A

Thank you for this comment. We corrected the incorrect reference in line 106 on p.5 (Fig. S2A) in the revised version.

(5) On line 315, the reference used is number 49, but there are 45 references, so it might be 39 instead.

Thank you for this comment. We corrected the incorrect reference in line 288 on p.12 "the DR274 plasmid (Hwang et al., 2013)" in the revised version.

Reviewer 2

We are grateful to Reviewer 2 for the useful suggestions that have helped us to considerably improve our paper. As indicated in the responses that follow, we have taken all these comments and suggestions into account in the revised version of our manuscript.

1/ rplf as an amputation induced gene.

The expression pattern by in situ is compatible with expression in leucocyte. However: (1)Additional marker should be used to demonstrate that the cells are indeed leucocytes (i.e Paredes et al dev biol, 2015) or qtify rplf in situ in WT versus crispr CSF1 tadpole.

Thank you for this comment. Accordingly, we performed *in situ* hybridization of *rfem.L/S* (the gene name *rplf* was changed to *rfem* in the revised manuscript, according to Reviewer 1's comment-7) in amputated tails of *csf1* knockdown (KD) and *tyrosinase* KD (*tyr* KD; as negative control, according to Reviewer 1's comment-1b) tadpoles and counted the number of *rfem*-expressing cells per section. We detected a significant reduction in the number of *rfem*-expressing cells in the tail stumps of *csf1* KD tadpoles compared with that of *tyr* KD tadpoles. We described the result in lines 37-39 on p.2 (Abstract) and lines 180-183 on p.8 (Results and Discussion) and added a new Fig. 4B in the revised version.

(2)The statement that an increase in rplf expressing cells upon amputation is observed need to be backed up by quantitation of in situ data. This is important in light of the fact that in scRNA-seq dataset while rplf is restricted to leucocyte cluster it does not seem to be expressed in a regeneration dependent manner (suppl fig 3). Maybe that the authors can also evaluate if rpfl expression level change in the in the leucocyte cluster in tail versus blastema?

Thank you for this comment. Accordingly, we performed *in situ* hybridization of *rfem.L/S* of intact and 1 days post amputation (dpa) tails of wild-type tadpoles and counted the number of *rfem*-expressing cells per section. We observed a significant increase in the number of *rfem*-expressing cells in 1 dpa tail stumps compared with that of intact tails. We described the result in lines 93-95 on p.4 and added a new Fig. 1E. Regarding evaluation of *rfem* expression levels in leukocytes, we did not think that we could isolate an adequate number of leukocytes to quantify *rfem*

© 2023. Published by The Company of Biologists under the terms of the Creative Commons Attribution License (https://creativecommons.org/licenses/by/4.0/).

expression with our methodologies within the limited timeframe for revision. We plan to proceed with the analysis in the future as the issue addresses important points to elucidate the Rfem function in the leukocytes.

2/ To address their initial question they need to perform crspr on IL11 and check if rplf is indeed not induced in leucocytes

Thank you for this comment. We think that the main scope of this article is the relation between *rfem* expression in leukocytes and tail regeneration, although the motivation for this study was the finding that *rfem* expression was downregulated in *il11* KD tadpoles. We described this context in lines 151-152 on p.7 and added a new Fig. S3E, which shows the expression levels of *rfem.L* and *rfem.S* in tail stumps at 48 h post amputation of *il11* KD and control tadpoles in the RNA-seq in a previous study (Tsujioka et al., 2017). We think that examination of the effect of *il11* on the expression of *rfem* in leukocytes would be outside the scope of this article. For clarification, we revised the sentence "We identified the *rplf*- expressing leukocytes as the hypothesized cell type that supports tail regeneration in response to IL-11 signaling …" in the Discussion part in the previous version, to "we identified *rfem.L/S* as genes required for tail regeneration …" in lines 198 on p.9 in the revised version.

3/As presented the crispr CSF1 KO is not very well linked to the rest of story. I would see it better as a tool to demonstrate that RFPL is expressed in leucocyte (see point 1). Indeed the role of leucocyte in regeneration is already documented (Aztekin et al, development 2020; Fukawaza et al development ,2009) so novelty here is not so strong. At least it should be put in perspective with these other studies.

Thank you for this comment. In the revision, we performed a cell-type specific rescue experiment as follows: We designed constructs that express *rfem.L* and *acgfp1* bicistronically, or only *acgfp1* under control of the zebrafish *mpeg1* promoter (*mpeg1:rfem* and *mpeg1:gfp* respectively) to perform macrophage-like cell-specific forced-expression of *rfem* in *rfem* KD tadpoles. The expression cassette was flanked by 2 I-*Sce*I meganuclease recognition sites to perform I-*Sce*I mediated transgenesis, which would facilitate expression by stably inserting into the genome, in addition to the free constructs remaining in the cells of the tadpole at the time of tail amputation. We observed scattered GFP-expressing cells in regenerating tails of tadpoles injected with the construct, suggesting that the construct worked as expected. Compared with control individuals (*rfem* KD individuals co-injected with *mpeg1:gfp*), the area and length of the regenerated tails in the *mpeg1:rfem*-rescued *rfem* KD individuals 7 dpa were significantly recovered, indicating that *rfem* expression in macrophage-like cells has an indispensable role in successful tail regeneration. We described the results in lines 39-41 on p.2 (Abstract) and lines 187-197 on p.8-9 (Results and Discussion) and added new Figs. 4C- F and S4H-K. This study identified *rfem* as a factor that assumes a regeneration-promoting function of macrophage-like cells; we think this is the novelty of the present study.

4/In general the crispr phenotypte should be better analysed/presented.

(1)-As mentioned by authors the crispr F0 assay shows a mosaic effect. The authors present for each crispr target 8 different experiments with only a subset showing effect (i.e 5/8 for the rplf crispr assay). I assume the reason for heterogeneity is difference in crispr efficiency, but there is no data to back this up. The regeneration assay is well suited to measure crispr efficiency from individual tadpoles (using amputated tail material), for example using a T7-Endonuclease I assay to quantify the percentage of heteroduplex, or using TIDE . This would be a more convincing way to link crispr efficiency to regeneration outcome.

Thank you for this comment. This comment is similar to that of Reviewer 1's comment-1a.

As described in our response to Reviewer 1's comment-1a, regarding *rfem*, *rfem.L/S* are expressed in a subset of leukocytes and it would thus be preferable that genotyping on genomic DNA of the leukocyte fraction isolated from each F0 crispant be used to link crispr efficiency with regeneration outcome; however, we did not think we could perform the experiment with the current methodologies within a reasonable timeframe. Instead, we tried to detect a correlation between the phenotype (the area of regenerated tail) and genotype using ICE analysis, a method

© 2023. Published by The Company of Biologists under the terms of the Creative Commons Attribution License (https://creativecommons.org/licenses/by/4.0/).

similar to TIDE, with genomic DNA of the peripheral blood cell (PBC) fraction isolated from each individual, but no correlation was detected, possibly due to the small proportion of the leukocyte subset in the PBC fraction. We described this result in lines 160-164 on p.7 and added a new Fig. S2I in the revised version. We did not think that the batch effect (differences between experiments) could be addressed with this method, and therefore we did not repeat the experiment and instead dealt with this issue in another way. The reviewer's concern is that our previous data of regeneration outcome assessments showed considerable differences between experiments. In the revised version, we measured the area and length of the regenerated tails to assess the regenerative capacity, according to the comments of Reviewers 1 and 2. We detected significantly reduced regenerative capacity in the *rfem* KD group with reproducibility; the results of all 3 experiments w showed a significant reduction. This method minimized the batch effects between experiments on the regenerative capacity assessment. We described this result in lines 124-125 on p.6, and added new Figs. 2D,E, and S2F-H in the revised version.

Regarding *csf1*, linking crispr efficiency with the regeneration outcome in the *csf1* KD experiment should be performed with genotyping on genomic DNA from a cell fraction that expresses *csf1*, but we have no methodologies to isolate the fraction. We utilized *csf1* to assess the relationship between *rfem* and macrophage-lineage cells by *csf1* KD and subsequent depletion of the *csf1*-dependent leukocytes; in the revised version, we performed forced expression of *rfem.L* in the macrophage-like cells of *rfem* KD individuals under control of the zebrafish *mpeg1* promoter, and found that the forced expression recovered regeneration outcomes of *rfem* KD individuals. This result strongly indicated that defects in tail regeneration in the *rfem* KD group were largely due to the loss of *rfem* function in macrophage-like cells. We described this result in lines 39-41 on p.2 (Abstract) and lines 187-197 on p.8-9 (Results and Discussion), and added new Figs. 4C-F and S4H-K in the revised version. We think that the result of the rescue experiment further strengthens our argument that the *rfem* expression in macrophage-lineage cells is required for tail regeneration, even without having linked crispr efficiency with the regeneration outcome of the *csf1* KD experiment, which is technically difficult for us.

We hope that our responses satisfactorily address the reviewer's concern.

(2)-Similarly it would be important to check that those tadpoles excluded from the analysis (abnormal) are not those showing the strongest crispr KD of rplf (indeed rplf KD show more abnormality than control)

Thank you for this comment. Accordingly, we compared the gene-editing efficiency at each gRNA target site of the genomic *rfem.L/S* locus in *rfem* KD individuals with developmental abnormalities and normally developed individuals using ICE analysis, and we detected no significant differences in the gene-editing efficiency between them, suggesting that the developmental abnormalities exhibited by some *rfem.L/S* KD individuals are not due to the *rfem.L/S* KD. Considering that *rfem.L/S* are rarely expressed in early development, we estimate that the effect of *rfem.L/S* KD on development is limited. We described this result in lines 114-119 on p.5, and added a new Fig. S2C in the revised version.

(3)-It is not clear why the assessment of regeneration is shown with percentage of imperfect/perfect. Why are the authors not using the more detailed RI index T7- Endonucléase lused in previous work from this group (i.e Fukawaza et al development ,2009)

Thank you for this comment. Accordingly, as described in our response to Reviewer

2's comment 4(1), we measured the area and length of the regenerated tails to assess the regenerative capacity. We detected a significantly reduced regenerative capacity in the *rfem* KD group with reproducibility; all 3 experiments we performed showed a significant reduction. This method minimized the batch effects between experiments on the regenerative capacity assessment. We described this result in lines 124-125 on p.5, and added new Fig. 2D, E, and S2F-H in the revised version.

© 2023. Published by The Company of Biologists under the terms of the Creative Commons Attribution License (https://creativecommons.org/licenses/by/4.0/).

Second decision letter

MS ID#: DEVELOP/2021/200467

MS TITLE: *rfem* expression in macrophage-like cells is required for tail regeneration in *Xenopus laevis* tadpole

AUTHORS: Momoko Deguchi, Taro Fukazawa, and Takeo Kubo

I have now received all the referees reports on the above manuscript, and have reached a decision. The referees' comments are appended below, or you can access them online: please go to BenchPress and click on the 'Manuscripts with Decisions' queue in the Author Area.

The overall evaluation is positive and we would like to publish a revised manuscript in Development, provided that the referees' comments can be satisfactorily addressed. Please attend to all of the reviewers' comments in your revised manuscript and detail them in your point-by-point response. If you do not agree with any of their criticisms or suggestions explain clearly why this is so. If it would be helpful, you are welcome to contact us to discuss your revision in greater detail. Please send us a point-by-point response indicating your plans for addressing the refereeâ€™s comments, and we will look over this and provide further guidance.

Reviewer 1

Advance summary and potential significance to field

N/A (revision assessment, see previous comments)

Comments for the author

The authors have made significant efforts towards addressing my comments, and the resulting manuscript is very much improved. In particular, I appreciate the rescue experiment, which considerably strengthens the main claim. There is a point which was not fully addressed, this being whether macrophages/monocyte themselves are reduced in the KD backgrounds (this is NOT directly addressed by doing rpfm ISH as the authors claim in their response, this needs addressing employing ISH against a more established macrophage specific marker). I think from the paper's perspective it is worth addressing as it adds to the biology. If this cannot be tackled, it should at least be discussed within the text.

Reviewer 2

Advance summary and potential significance to field

The authors have answer most of concerns from initial assesment.
Howhever a few concerns remain as well as a possible important issue with a new important piece of data provided. See details below

Comments for the author

I have two reservations concerning authors revisions:
1/ They now provide quantification of in situ data to back up some of their claims (i.e 4B and 1E). However data provided seems to be on very limited sample size (3 to 4 tadpoles). This are presumably from a single experiment. I would be more comfortable with stat test carried out on independent experiments.
2/A very important new piece of data is the rescue of rfem KD by an mpeg-rfem construct. I am wondering whether there could possibly be a major flaw in rescue design? Indeed it seems to me that the rfem crispr can affect both the genomic and the rescue construct copy of rfem. So is the observed phenotype rescue really a rescue of genomic rfem KB by mpeg driven rfem or is it

© 2023. Published by The Company of Biologists under the terms of the Creative Commons Attribution License (https://creativecommons.org/licenses/by/4.0/).

inhibition of genomic rfem KB by the rescue construct? In which case the experiment does not support a role for macrophage-like cell expression of rfem in regeneration.
Can the authors alleviate these concerns?
3/ Also I think it would be beneficial to show data as dot plots rather than bar plot when parameter from many tadpoles are plotted (i.e plots such as that in 2E S2F_H etc..)

Second revision

Author response to reviewers' comments

Reviewer 1

We would like to thank Reviewer 1 for the suggestion that has helped us considerably improve our paper. We have addressed this suggestion in the revised version of our manuscript, as indicated in the response below.

There is a point which was not fully addressed, this being whether macrophages/monocyte themselves are reduced in the KD backgrounds (this is NOT directly addressed by doing rpfm ISH as the authors claim in their response, this needs addressing employing ISH against a more established macrophage specific marker). I think from the paper's perspective it is worth addressing as it adds to the biology. If this cannot be tackled, it should at least be discussed within the text.

Thank you for this comment. Accordingly, we performed *in situ* hybridization against *c1qa.L* in *csf1* KD tadpoles because *C1q* is expressed in macrophages in mice and we previously observed a significant reduction in the *c1qa.L* expression level in *csf1* KD tadpoles by qRT-PCR (Figure S4E). We observed a slight, but not statistically significant, reduction in the number of *c1qa.L*-expressing cells in the amputated tails of *csf1* KD tadpoles. The expression level of *c1qa.L* is likely to be remarkably lower than that of *rfem* (based on the range of the vertical axis of Fig. 4A [*rfem*] and S4E [*c1qa.L*], the expression level of *c1qa.L* is estimated to be approximately 1/10 of that of *rfem*), making it difficult detect all *c1qa.L*-expressing cells and thus a significant reduction in their number. We described these results and discussion in lines 177–182 on p.8 and added a new Fig. S4F.

Current results of the *in situ* hybridization against *c1qa.L* did not provide strong support for macrophage depletion in *csf1* KD tadpoles, although with regard to *rfem*, we observed a significant reduction in both the expression and number of expressing cells in the tail stumps of *csf1* KD tadpoles, suggesting that *csf1* KD depleted *rfem*-expressing cells. Therefore, we revised the sentence "These results suggested that *csf1* KD depleted the *csf1* dependent leukocytes, including *rfem*-expressing cells" in the previous version to "… that *csf1* KD depleted the *rfem*-expressing leukocytes" in lines 176–177 on p.8 of the revised manuscript.

Reviewer 2

We would like to thank Reviewer 2 for the suggestions that have helped us considerably improve our paper. We have addressed these suggestions in the revised version of our manuscript, as indicated in the response below.

I have two reservations concerning authors revisions:

1/ They now provide quantification of in situ data to back up some of their claims (i.e 4B and 1E). However data provided seems to be on very limited sample size (3 to 4 tadpoles). This are presumably from a single experiment. I would be more comfortable with stat test carried out on independent experiments.

© 2023. Published by The Company of Biologists under the terms of the Creative Commons Attribution License (https://creativecommons.org/licenses/by/4.0/).

Thank you for this comment. Accordingly, we performed additional *in situ* experiments to increase the sample sizes and obtained similar results to the previous data. The sample sizes of 3–4 individuals in the previous version were increased 7–9 individuals in the revised version. We replaced Fig. 1E and 4B in the previous version with new Fig. 1E and 4B, and revised the figure legends. The data are shown as dot plots, according to Reviewer 2's comment-3.

2/A very important new piece of data is the rescue of rfem KD by an mpeg-rfem construct. I am wondering whether there could possibly be a major flaw in rescue design? Indeed it seems to me that the rfem crispr can affect both the genomic and the rescue construct copy of rfem. So is the observed phenotype rescue really a rescue of genomic rfem KB by mpeg driven rfem or is it inhibition of genomic rfem KB by the rescue construct? In which case the experiment does not support a role for macrophage-like cell expression of rfem in regeneration.

Can the authors alleviate these concerns?

Thank you for this comment. In the rescue construct, we introduced synonymous substitutions into the *rfem.L* sequence at guide RNA target sites to prevent gene editing of the rescue construct. This information was provided in the Materials and Methods section (lines 399-401 on p.17). To improve the readability of the manuscript, we added this information to the figure legend of Fig. S4I, which describes the structure of the rescue construct. We consider that *mpeg1*-dependent *rfem.L* expression was successful and that the results of the rescue experiment support a role for *rfem* in the macrophage- like cells during regeneration.

3/ Also I think it would be beneficial to show data as dot plots rather than bar plot when parameter from many tadpoles are plotted (i.e plots such as that in 2E S2F_H etc..)

Thank you for this comment. Accordingly, we replaced Fig. 1E, 2E, 4B, 4F, S2H, S4J, and K in the previous version with new Fig. 1E, 2E, 4B, 4F, S2H, S4K, and L, in which the data are now shown as dot plots.

Third decision letter

MS ID#: DEVELOP/2021/200467

MS TITLE: *rfem* expression in macrophage-like cells is required for tail regeneration in *Xenopus laevis* tadpole

AUTHORS: Momoko Deguchi, Taro Fukazawa, and Takeo Kubo
ARTICLE TYPE: Research Report

I am happy to tell you that your manuscript has been accepted for publication in Development, pending our standard ethics checks.

Reviewer 1

Advance summary and potential significance to field

n/a

Comments for the author

My point/s have been satisfactorily addressed.
I recommend the publication of this interesting work.

© 2023. Published by The Company of Biologists under the terms of the Creative Commons Attribution License (https://creativecommons.org/licenses/by/4.0/).

Reviewer 2

Advance summary and potential significance to field

Using a combinatation of knockdown and cell type specific rescue experiements the authors show that the expression of a new gene they named "regeneration factors expressed on myeloid (rfem)" in macrophage-like cells is required for tail regeneration.

Comments for the author

Authors have addressed my concerns concerning sample size/data representation and design of the rescue experiment

© 2023. Published by The Company of Biologists under the terms of the Creative Commons Attribution License (https://creativecommons.org/licenses/by/4.0/).
